# A comparative genomics multitool for scientific discovery and conservation

**DOI:** 10.1038/s41586-020-2876-6

**Published:** 2020-11-11

**Authors:** Zoonomia Consortium, Diane P. Genereux, Aitor Serres, Joel Armstrong, Jeremy Johnson, Voichita D. Marinescu, Eva Murén, David Juan, Gill Bejerano, Nicholas R. Casewell, Leona G. Chemnick, Joana Damas, Federica Di Palma, Mark Diekhans, Ian T. Fiddes, Manuel Garber, Vadim N. Gladyshev, Linda Goodman, Wilfried Haerty, Marlys L. Houck, Robert Hubley, Teemu Kivioja, Klaus-Peter Koepfli, Lukas F. K. Kuderna, Eric S. Lander, Jennifer R. S. Meadows, William J. Murphy, Will Nash, Hyun Ji Noh, Martin Nweeia, Andreas R. Pfenning, Katherine S. Pollard, David A. Ray, Beth Shapiro, Arian F. A. Smit, Mark S. Springer, Cynthia C. Steiner, Ross Swofford, Jussi Taipale, Emma C. Teeling, Jason Turner-Maier, Jessica Alfoldi, Bruce Birren, Oliver A. Ryder, Harris A. Lewin, Benedict Paten, Tomas Marques-Bonet, Kerstin Lindblad-Toh, Elinor K. Karlsson

**Affiliations:** 1 Broad Institute of MIT and Harvard, Cambridge, MA USA; 2 Institute of Evolutionary Biology (UPF-CSIC), PRBB, Barcelona, Spain; 3 UC Santa Cruz Genomics Institute, University of California Santa Cruz, Santa Cruz, CA USA; 4 Science for Life Laboratory, Department of Medical Biochemistry and Microbiology, Uppsala University, Uppsala, Sweden; 5 Department of Biomedical Data Science, Stanford University, Stanford, CA USA; 6 Department of Computer Science, Stanford University, Stanford, CA USA; 7 Department of Developmental Biology, Stanford University, Stanford, CA USA; 8 Department of Pediatrics, Stanford University, Stanford, CA USA; 9 Centre for Snakebite Research and Interventions, Liverpool School of Tropical Medicine, Liverpool, UK; 10 San Diego Zoo Global, Beckman Center for Conservation Research, San Diego, CA USA; 11 The UC Davis Genome Center, University of California, Davis, Davis, CA USA; 12 Department of Biological Sciences, University of East Anglia, Norwich, UK; 13 Earlham Institute, Norwich, UK; 14 Program in Bioinformatics and Integrative Biology, University of Massachusetts Medical School, Worcester, MA USA; 15 Brigham and Women’s Hospital, Harvard Medical School, Boston, MA USA; 16 Fauna Bio Incorporated, Emeryville, CA USA; 17 Institute for Systems Biology, Seattle, WA USA; 18 Department of Biochemistry, University of Cambridge, Cambridge, UK; 19 Applied Tumor Genomics Research Program, University of Helsinki, Helsinki, Finland; 20 Smithsonian-Mason School of Conservation, Front Royal, VA USA; 21 Department of Biology, MIT, Cambridge, MA USA; 22 Department of Systems Biology, Harvard Medical School, Boston, MA USA; 23 Veterinary Integrative Biosciences, Texas A&M University, College Station, TX USA; 24 Marine Mammal Program, Smithsonian Institution, Washington, DC USA; 25 Restorative Dentistry and Biomaterials Sciences, Harvard School of Dental Medicine, Boston, MA USA; 26 School of Dental Medicine, Case Western Reserve University, Cleveland, OH USA; 27 Carnegie Mellon University, School of Computer Science, Department of Computational Biology, Pittsburgh, PA USA; 28 Chan Zuckerberg Biohub, San Francisco, CA USA; 29 Gladstone Institutes, San Francisco, CA USA; 30 Department of Epidemiology and Biostatistics, Institute for Computational Health Sciences and Institute for Human Genetics, University of California, San Francisco, San Francisco, CA USA; 31 Department of Biological Sciences, Texas Tech University, Lubbock, TX USA; 32 Department of Ecology and Evolutionary Biology, University of California, Santa Cruz, Santa Cruz, CA USA; 33 Howard Hughes Medical Institute, University of California Santa Cruz, Santa Cruz, CA USA; 34 Department of Evolution, Ecology and Organismal Biology, University of California, Riverside, Riverside, CA USA; 35 Department of Medical Biochemistry and Biophysics, Karolinska Institutet, Stockholm, Sweden; 36 School of Biology and Environmental Science, University College Dublin, Dublin, Ireland; 37 Department of Evolution, Behavior, and Ecology, Division of Biology, University of California, San Diego, La Jolla, CA USA; 38 Department of Evolution and Ecology, University of California, Davis, Davis, CA USA; 39 Catalan Institution of Research and Advanced Studies (ICREA), Barcelona, Spain; 40 Institut Català de Paleontologia Miquel Crusafont, Universitat Autònoma de Barcelona, Barcelona, Spain; 41 CNAG-CRG, Centre for Genomic Regulation (CRG), Barcelona Institute of Science and Technology (BIST), Barcelona, Spain; 42 Program in Molecular Medicine, University of Massachusetts Medical School, Worcester, MA USA

**Keywords:** Genome informatics, Ecological genetics, Evolutionary genetics, Phylogenetics, Comparative genomics

## Abstract

The Zoonomia Project is investigating the genomics of shared and specialized traits in eutherian mammals. Here we provide genome assemblies for 131 species, of which all but 9 are previously uncharacterized, and describe a whole-genome alignment of 240 species of considerable phylogenetic diversity, comprising representatives from more than 80% of mammalian families. We find that regions of reduced genetic diversity are more abundant in species at a high risk of extinction, discern signals of evolutionary selection at high resolution and provide insights from individual reference genomes. By prioritizing phylogenetic diversity and making data available quickly and without restriction, the Zoonomia Project aims to support biological discovery, medical research and the conservation of biodiversity.

## Abstract (web summary)

A whole-genome alignment of 240 phylogenetically diverse species of eutherian mammal—including 131 previously uncharacterized species—from the Zoonomia Project provides data that support biological discovery, medical research and conservation.

## Main

The genomics revolution is enabling advances not only in medical research^1^, but also in basic biology^2^ and in the conservation of biodiversity, where genomic tools have helped to apprehend poachers^3^ and to protect endangered populations^4^. However, we have only a limited ability to predict which genomic variants lead to changes in organism-level phenotypes, such as increased disease risk—a task that, in humans, is complicated by the sheer size of the genome (about three billion nucleotides)^5^.

Comparative genomics can address this challenge by identifying nucleotide positions that have remained unchanged across millions of years of evolution^6^ (suggesting that changes at these positions will negatively affect fitness), focusing the search for disease-causing variants. In 2011, the 29 Mammals Project^7^ identified 12-base-pair (bp) regions of evolutionary constraint that in total comprise 4.2% of the genome, by measuring sequence conservation in humans plus 28 other mammals. These regions proved to be more enriched for the heritability of complex diseases than any other functional mark, including coding status^8^. By expanding the number of species and making an alignment that is independent of any single reference genome, the Zoonomia Project was designed to detect evolutionary constraint in the eutherian lineage at increased resolution, and to provide genomic resources for over 130 previously uncharacterized species.

## Designing a comparative-genomics multitool

**Fig. 1:**
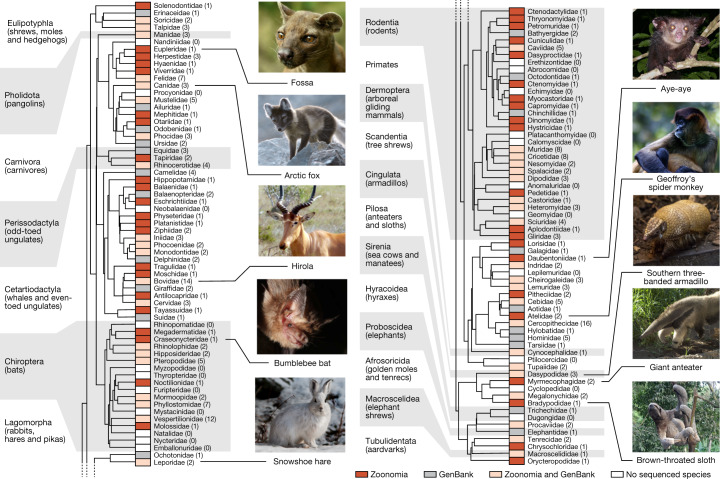
The Zoonomia Project brings the fraction of eutherian families that are represented by at least one assembly to 83%. Phylogenetic tree of the mammalian families in the Zoonomia Project alignment, including both our new assemblies and all other high-quality mammalian genomes publicly available in GenBank when we started the alignment (March 2018) (Supplementary Table 2). Tree topology is based on data from TimeTree (www.timetree.org)^47^. Existing taxonomic classifications recognize a total of 127 extant families of eutherian mammal^48^, including 43 families that were not previously represented in GenBank (red boxes) and 41 families with additional representative genome assemblies (pink boxes). Of the remaining families, 21 had GenBank genome assemblies but no Zoonomia Project assembly (grey boxes) and 22 had no representative genome assembly (white boxes). Parenthetical numbers indicate the number of species with genome assemblies in a given family. Image credits: fossa, Bertal/Wikimedia (CC BY-SA); Arctic fox, Michael Haferkamp/Wikimedia (CC BY-SA); hirola, JRProbert/Wikimedia (CC BY-SA); bumblebee bat, Sébastien J. Puechmaille (CC BY-SA); snowshoe hare, Denali National Park and Preserve/Wikimedia (public domain); aye-aye, Tom Junek/Wikimedia (CC BY-SA); Geoffroy’s spider monkey, Patrick Gijsbers/Wikimedia (CC BY-SA); southern three-banded armadillo, Hedwig Storch/Wikimedia (CC BY-SA); giant anteater, Graham Hughes/Wikimedia (CC BY-SA); brown-throated sloth, Dick Culbert from Gibsons, B.C., Canada/Wikimedia (CC BY).

When selecting species, we sought to maximize evolutionary branch length, to include at least one species from each eutherian family, and to prioritize species of medical, biological or biodiversity conservation interest. Our assemblies increase the percentage of eutherian families with a representative genome from 49% to 82%, and include 9 species that are the sole extant member of their family and 7 species that are critically endangered^9^ (Fig. 1): the Mexican howler monkey (*Alouatta palliata mexicana*), hirola (*Beatragus hunteri*), Russian saiga (*Saiga tatarica tatarica*), social tuco-tuco (*Ctenomys sociabilis*), indri (*Indri indri*), northern white rhinoceros (*Ceratotherium simum cottoni*) and black rhinoceros (*Diceros bicornis*).

We collaborated with 28 institutions to collect samples, nearly half (47%) of which were provided by The Frozen Zoo of San Diego Zoo Global (Supplementary Table 1). Since 1975, The Frozen Zoo has stored renewable cell cultures for about 10,000 vertebrate animals that represent over 1,100 taxa, including more than 200 species that are classified as vulnerable, endangered, critically endangered or extinct by the International Union for Conservation of Nature (IUCN)^10^. For 36 target species we were unable to acquire a DNA sample of sufficient quality, even though our requirements were modest (Methods), which highlights a major impediment to expanding the phylogenetic diversity of genomics.

**Extended Data Fig. 1:**
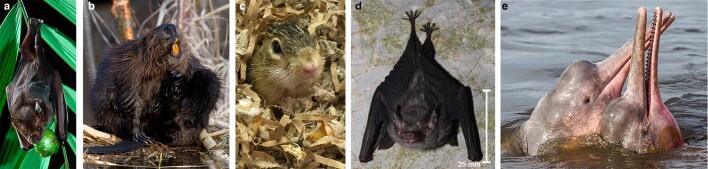
Notable traits in non-human mammals. Sequences from species with notable phenotypes can inform human medicine, basic biology and biodiversity conservation, but sample collection can be challenging. **a**, The Jamaican fruit bat (*Artibeus jamaicensis*) maintains constant blood glucose across intervals of fruit-eating and fasting^66^, achieving homeostasis to a degree that is unknown in the treatment of human diabetes. **b**, The North American beaver (*Castor canadensis*) avoids tooth decay by incorporating iron rather than magnesium into tooth enamel, which yields an orange hue^67^. **c**, The thirteen-lined ground squirrel (*Ictidomys tridecemlineatus*) prepares for hibernation by rapidly increasing the thermogenic activity of brown fat^68^, a process that—in humans—is connected to improved glucose homeostasis and insulin sensitivity^69–71^. **d**, The tiny bumblebee bat (*Craseonycteris thonglongyai*) is among the smallest of mammals, making it a sparse source of DNA. **e**, The remote habitat of the very rare Amazon River dolphin (*Inia geoffrensis*) precludes collection of the high-molecular weight DNA. Image sources: Merlin D. Tuttle/Science Source (**a**); Stephen J. Krasemann/Science Source (**b**); Allyson Hindle (**c**); Sébastien J. Puechmaille (CC BY-SA) (**d**); M. Watson/Science Source (**e**).

**Extended Data Fig. 2:**
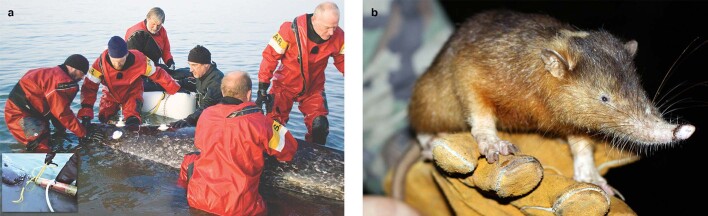
Sample collection can be challenging, and sequencing methods must be selected to handle the sample quality. To enable the inclusion of species from across the eutherian tree (including from the 50% of mammalian families not represented in existing genome databases), the Zoonomia Project needed sequencing and assembly methods that produce reliable data from DNA collected in remote locations, sometimes in only modest quantities and often without benefit of cold chains for transport. **a**, For the marine species such as the narwhal (*Monodon monoceros*), simply accessing an individual in the wild can prove challenging. For example, to sample DNA from the near-threatened narwhal, M.N. and Inuit guide D. Angnatsiak camped on the edge of an ice floe between Pond Inlet and Bylot Island, at the northeastern tip of Baffin Island. After a narwhal was collected by Inuit hunters as part of an annual hunt, hours of flensing were necessary for the collection of tissue samples. From left to right, F. McCann, H. C. Schmidt, F. Eichmiller, M.N., J. Orr (facing backward) and J. Orr (standing). **b**, For endangered species such as the Hispaniolan solenodon (*S. paradoxus*), sample collection must be designed to minimize stress to the individual, limiting the amount of DNA that can be collected^22^. To collect DNA from the endangered solenodon without imposing stress on individuals in the wild, N.R.C. turned to the world’s only captive solenodons, which are housed off-exhibit at ZOODOM in the Dominican Republic. With help from veterinarians at the zoo, N.R.C. collected a small amount of blood from the rugged tail of the solenodon. Narwhal photograph by G. Freund, and courtesy of M.N. Solenodon photograph courtesy of L. Emery.

We used two complementary approaches to generate genome assemblies (Extended Data Table 1). First, for 131 genomes we generated assemblies by performing a single lane of sequencing (2× 250-bp reads) on PCR-free libraries and assembling with DISCOVAR de novo^11^ (referred to here as ‘DISCOVAR assemblies’). This method does not require intact cells and uses less than two micrograms of medium-quality DNA (most fragments are over 5 kilobases (kb) in size), which allowed us to include species that are difficult to access (Extended Data Figs. 1, 2) while achieving ‘contiguous sequences constructed from overlapping short reads’ (contig) lengths comparable to those of existing assemblies (median contig N50 of 46.8 kb, compared to 47.9 kb for Refseq genome assemblies).

For nine DISCOVAR assemblies and one pre-existing assembly (the lesser hedgehog tenrec (*Echinops telfairi*)), we increased contiguity 200-fold (the median scaffold length increased from 90.5 kb to 18.5 megabases (Mb)) through proximity ligation, which uses chromatin interaction data to capture the physical relationships among genomic regions^12^. Unlike short-contiguity genomes, these assemblies capture structural changes such as chromosomal rearrangements^13^. The upgraded assemblies increase the number of eutherian orders that are represented by a long-range assembly (contig N50 > 20 kb and scaffold N50 > 10 Mb) from 12 to 18 (out of 19). We are working on upgrading the assembly of the large treeshrew (*Tupaia tana*) for the remaining order (Scandentia).

## Comparative power of 240 species

The Zoonomia alignment includes 120 newly generated assemblies and 121 existing assemblies, representing a total of 240 species (the dataset includes assemblies for two different dogs) and spanning about 110 million years of mammalian evolution (Supplementary Table 2). With a total evolutionary branch length of 16.6 substitutions per site, we expect only 191 positions in the human genome (0.000006%) to be identical across the aligned species owing to chance (false positives) rather than evolutionary constraint (Extended Data Table 2). We applied this same calculation to data from The Exome Aggregation Consortium (ExAC)—who analysed exomes for 60,706 humans^14^—and estimated that 88% of positions would be expected to have no variation. This illustrates the potential for relatively small cross-species datasets to inform human genetic studies—even for diseases driven by high-penetrance coding mutations, for which ExAC data are optimally powered^15^.

## Biological insights from additional assemblies

The scope and species diversity in the Zoonomia Project supports evolutionary studies in many lineages. Previously published papers (discussed in the subsections below), and the demonstrated utility of existing comparative genomics resources^16,17^, illustrate the benefits of making newly generated genome assemblies and alignments accessible to all researchers without restrictions on use.

### Speciation

Comparing our assembly for the endangered Mexican howler monkey (*Alouatta palliata mexicana*, a subspecies of the mantled howler monkey) with the Guatemalan black howler monkey (*Alouatta pigra*)—which has a neighbouring range—suggests that different forms of selection shape the reproductive isolation of the two species^18^. Initial divergence in allopatry was followed by positive selection on postzygotic isolating mechanisms, which offers empirical support for a speciation process that was first outlined by Dobzhansky in 1935^19^.

### Protection from cancer

Using our assembly for the capybara (*Hydrochoerus hydrochaeris*) (a giant rodent), a previous publication^20^ has identified positive selection on anti-cancer pathways, echoing previous reports^21^ that other large mammal species—the African and Asian elephants (*Loxodonta africana* and *Elephas maximus indicus*, respectively) —carry extra copies (retrogenes) of the tumour-suppressor gene *TP53*. This offers a possible resolution to Peto’s paradox—the observation that cancer in large mammals is rarer than expected—and could reveal anti-cancer mechanisms.

### Convergent evolution of venom

A previous publication^22^ has used our assembly for the Hispaniolan solenodon (*Solenodon paradoxus*) (Extended Data Fig. 2) to investigate venom production—a trait that is found in only a few eutherian lineages, including shrews and solenodons. They identified paralogous copies of a kallikrein 1 serine protease gene (*KLK1*) that together encode solenodon venom, and showed that the *KLK1* gene was independently co-opted for venom production in both solenodons and shrews, in an example of molecular convergence.

### Informing biodiversity conservation strategies

A previous analysis^23^ of our giant otter (*Pteronura brasiliensis*) assembly found low diversity and an elevated burden of putatively deleterious genetic variants, consistent with the recent population decline of this species through overhunting and habitat loss. The giant otter had fewer putatively deleterious variants than either the southern or northern sea otter (*Enhydra lutris nereis* and *E. lutris kenyoni*, respectively), which suggests that it has highest potential for recovery among these species if populations are protected.

### Rapid assessment of species infection risk

Using the Zoonomia alignment and public genomic data from hundreds of other vertebrates, a previous publication^24^ compared the structure of ACE2—the receptor for severe acute respiratory syndrome coronavirus 2 (SARS-CoV-2), the causative agent of coronavirus disease 2019 (COVID-19)—and identified 47 mammals that have a high or very high likelihood of being virus reservoirs, intermediate hosts or good model organisms for the study of COVID-19, and detected positive selection in the ACE2 receptor-binding domain that is specific to bats.

## Genetic diversity and extinction risk

We next asked whether a reference genome from a single individual can help to identify populations with low genetic diversity to prioritize in efforts to conserve biodiversity. Diversity metrics reflect demographic history^25,26^, and heterozygosity is lower in threatened species^27^. This analysis was feasible because we used a single sequencing and assembly protocol for all DISCOVAR assemblies, which minimized variation in accuracy, completeness and contiguity due to the sequencing technology and the assembly process that would otherwise confound species comparisons.

We estimated genetic diversity for 130 of our DISCOVAR assemblies, each of which represented a different species (Supplementary Table 3). Four of these estimates failed during analysis. For the remaining 126 DISCOVAR assemblies, we calculated 2 metrics: (1) the fraction of sites at which the sequenced individual is heterozygous (overall heterozygosity); and (2) the proportion of the genome that resides in an extended region without any variation (segments of homozygosity (SoH)). The SoH measurement is designed for short-contiguity assemblies, in which scaffolds are potentially shorter than runs of homozygosity. Overall, heterozygosity and SoH values are correlated (Pearson correlation *r* = −0.56, *P* = 1.8 × 10^−9^, *n* = 98). Although overall heterozygosity is correlated with contig N50 values (Pearson correlation *r*_het_ = −0.39, *P*_het_ = 4 × 10^−5^, *n*_het_ = 105) (probably owing to the difficulty of assembling more heterozygous genomes^28^), SoH values are not (Pearson correlation *r*_SoH_ = 0.09, *P*_SoH_ = 0.38, *n*_SoH_ = 98). Overall heterozygosity and SoH values are highly correlated between the lower- and high-contiguity versions of the upgraded assemblies (Pearson correlation *r*_het_ = 0.999, *P*_het_ = 5 × 10^−7^, *n*_het_ = 7; *r*_SoH_ = 0.996, *P*_SoH_ = 1.4 × 10^−6^, *n*_SoH_ = 7).

**Fig. 2:**
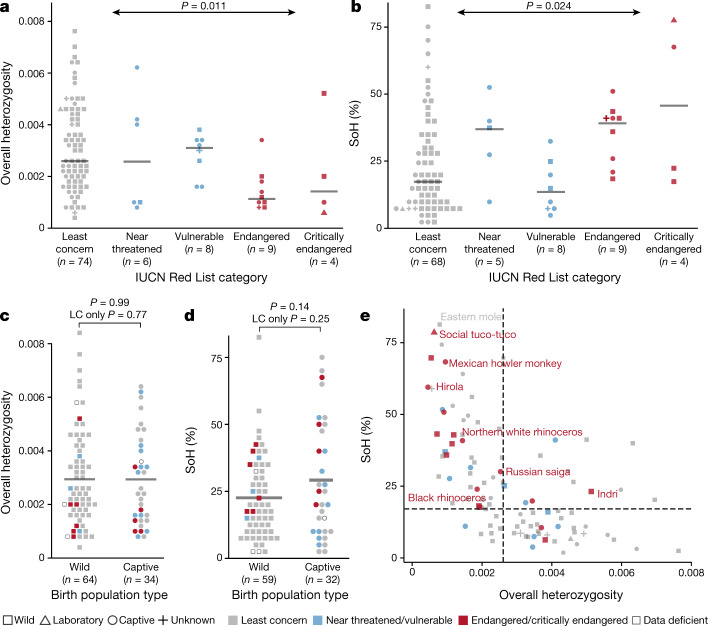
Genetic diversity varies across IUCN conservation categories. **a**, **b**, Heterozygosity declines (**a**) and SoH value increases (**b**) with the level of concern for species conservation, as assessed by IUCN conservation categories. Horizontal grey lines indicate median. **c**, **d**, Comparing individuals sampled from wild and captive populations, we saw no statistically significant difference (independent samples *t*-test) in overall heterozygosity (**c**) or per cent SoH (**d**), with similar means (horizontal grey lines) between types of birth population. In **a**–**d**, there was a total of 105 species, with *n* for each tested category indicated on the *x* axis. Statistical tests were two-sided. LC, least concern. **e**, Overall heterozygosity and SoH values for all genomes analysed (including those with high allelic balance ratio; *n* = 124 species), with median SoH (17.1%, horizontal dashed line) and median overall heterozygosity (0.0026, vertical dashed line) for species categorized as least concern. Values for individuals from the seven critically endangered species are shown in red. Source data

Genomic diversity varies significantly among species in different IUCN conservation categories, as measured by overall heterozygosity (Fig. 2a) and SoH values (Fig. 2b). SoH values increase (*P* = 0.024, *R*^2^ = 0.055, *n* = 94) with increasing levels of conservation concern, whereas heterozygosity decreases (*P* = 0.011, *R*^2^ = 0.064, *n* = 101). There is no significant difference between wild and captive populations in overall heterozygosity (Fig. 2c) or SoH values (Fig. 2d).

Unusual diversity values can suggest particular population demographics, although data from more than a single individual are needed to confirm these inferences. All seven critically endangered species have SoH values that are higher than the median for species categorized as of least concern (Fig. 2e). The genomes with the lowest heterozygosity and highest SoH values were the social tuco-tuco (heterozygosity = 0.00063 and SoH = 78.7%), which was sampled from a small laboratory colony with only 12 founders^29^, and the eastern mole (*Scalopus aquaticus*) (heterozygosity = 0.0008 and SoH = 81.3%), which was supplied by a professional mole catcher and was probably from a population that had experienced a bottleneck owing to pest control measures.

The correlation between diversity metrics and IUCN category is not explained by other species-level phenotypes. For species of least concern (*n* = 75), we assessed 21 phenotypes that are catalogued in the PanTHERIA^30^ database for correlation with heterozygosity or SoH values. The most significant was between SoH value and litter size, a trait that has previously been shown to predict extinction risk^31^ (*P*_SoH_ = 0.02), but none is significant after Bonferroni correction (Extended Data Table 3).

Our inference that diversity trends lower in species at a higher risk of extinction comes from a small fraction (2.6%) of threatened mammals^9^. Whether this is a direct correlation with extinction risk or arises from an association between diversity and species-level phenotypes such as litter size, it suggests that valuable information can be gleaned from sequencing only a single individual. Should this pattern prove robust across more species, diversity metrics from a single reference genome could help to identify populations that are at risk—even when few species-level phenotypes are documented—and to prioritize species for follow-up at the population level.

## Resources for biodiversity conservation

For each genome assembly, we catalogued all high-confidence variant sites (http://broad.io/variants) to support the design of cost-effective and accurate genetic assays that are usable even when the sample quality is low^32^; such assays are often preferable to designing expensive custom tools, relying on tools from related species or sequencing random regions^33^. The reference genomes themselves support the development of technologies such as using gene drives to control invasive species or pursuing ‘de-extinction’ through cloning and genetic engineering^34^.

Our genomes have two notable limitations. We sequenced only a single individual for each species, which is insufficient for studying population origins, population structure and recent demographic events^35,36^, and the shorter contiguity of our assemblies prevented us from analysing runs of homozygosity^26^. This highlights a dilemma that faces all large-scale genomics initiatives: determining when the value of sequencing additional individuals exceeds the value of improving the reference genome itself.

## Whole-genome alignment

**Fig. 3:**
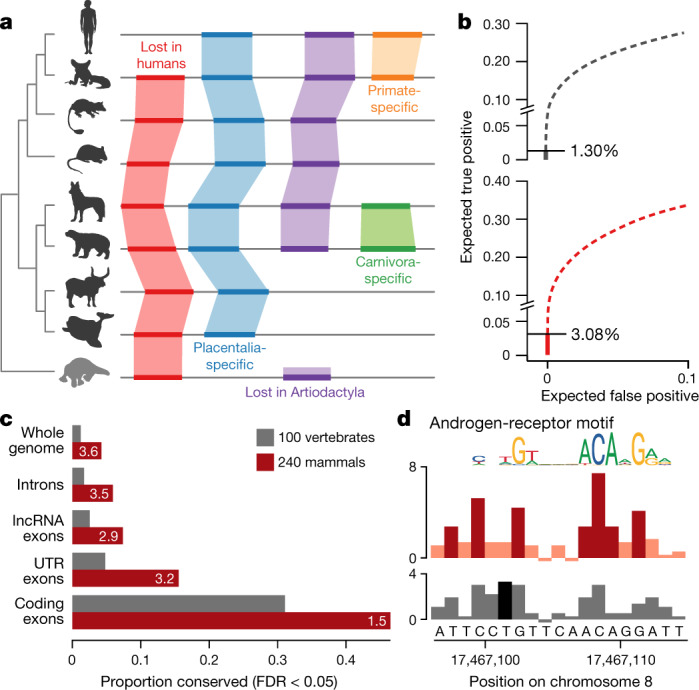
The Zoonomia alignment doubles the fraction of the human genome predicted to be under purifying selection at single-base-pair resolution. **a**, Cactus alignments are reference-genome-free, enabling the detection of sequence that is absent from human (red) or other clades (purple), lineage-specific innovations (orange and green) and eutherian-mammal-specific sequence (blue). **b**, We compared phyloP predictions of conserved positions for a widely used 100-vertebrate alignment (*n* = 100 vertebrate species) (grey) to the Zoonomia alignment (*n* = 240 eutherian species) (red). The cumulative portion of the genome expected to be covered by true- versus false-positive calls is shown, starting from the highest confidence calls (solid line) and proceeding to calls with lower confidence (dashed lines); the horizontal line indicates the point at which the confidence level drops below an expected FDR of 0.05 (two-sided). **c**, A higher proportion of functionally annotated bases are detected as highly conserved (FDR < 0.05) in the Zoonomia alignment (red) than the 100-vertebrate alignment (grey), most notably in non-coding regions. lncRNA, long non-coding RNA; UTR, untranslated region. **d**, At a putative androgen-receptor binding site, phyloP scores predict that seven bases are under purifying selection (FDR < 0.05, two-sided) in the Zoonomia alignment (dark red), peaking in positions with the most information content in the androgen receptor JASPAR^49^ motif, compared to one (dark grey) in the 100-vertebrate alignment, with scores at FDR > 0.05 shown in light red (top) and light grey (bottom).

We aligned the genomes of 240 species (our assemblies and other mammalian genomes that were released when we started the alignment) as part of a 600-way pan-amniote alignment using the Cactus alignment software^37^ (Supplementary Table 2). Rather than aligning to a single anchor genome, Cactus infers an ancestral genome for each pair of assemblies (Fig. 3a). Consistent with our predictions, we have increased power to detect sequence constraint at individual bases relative to previous studies^7,38^. We detect 3.1% of bases in the human genome to be under purifying selection in the eutherian lineage (false-discovery rate (FDR) < 5%), without using windowing or other means to integrate contextual information across neighbouring bases. This is more than double the number from the largest previous 100-vertebrate alignment^38^ (Fig. 3b), with improvements being most notable in the non-coding sequence (Fig. 3c) and in the increased resolution of individual features (Fig. 3d). This represents a substantial proportion—but not all—of the 5 to 8% of the human genome that has previously been suggested to be under purifying selection^7,39^.

## Next steps

Using our alignment of 240 mammalian genomes, we are pursuing four key strategies of analysis. First, we aim to provide the largest eutherian phylogeny based on nuclear genomes by building a comprehensive phylogeny and time tree, including trees partitioned by functional annotations, mode of inheritance and long-term recombination rates. Second, we will produce a detailed map of evolutionary constraint, identifying highly conserved genomic regions, regions under accelerated evolution in particular lineages and changes that probably affect phenotype, leveraging functional data from ENCODE^40^, GTEx^41^ and the Human Cell Atlas^42^. Third, we will use genotype–phenotype correlations to investigate patterns of constraint in regions associated with disease in humans, identify patterns of convergent adaptive evolution^2^ and apply a forward genomics strategy to link functional elements to traits. Finally, we will explore the evolution of genome structure by mapping syntenic regions between genomes, identifying evolutionary breakpoints and characterizing the repeat landscape.

## Conclusion

The Zoonomia Project has captured mammalian diversity at a high resolution, and is among the first of many projects that are underway to sequence, catalogue and characterize whole branches of the eukaryotic biodiversity of the Earth. On the basis of our experience, we propose the following principles for realizing the full value of large-scale comparative genomics.

First, we should prioritize sample collection. We must support field researchers who collect samples and understand species ecology and behaviour, develop strategies for sample collection that do not rely on bulky laboratory equipment or cold chains, develop technology for using non-invasive types of sampling and establish more repositories of renewable cell cultures^10^.

Second, we need accessible and scalable tools for computational analysis. Few research groups have access to the computational resources necessary for work with massive genomic datasets. We must address the shortage of skilled computational scientists, and design software and data-storage systems that make powerful computational pipelines accessible to all researchers.

Finally, we should promote rapid data-sharing. Data embargoes must not be permitted to delay analyses that directly benefit the conservation of endangered species, human health or progress in basic science. Genomic data should be shared as quickly as possible and without restrictions on use.

Numerous large-scale genome-sequencing efforts are now underway, including the Earth BioGenome Project^43^, Genome 10K^44^, the Vertebrate Genomes Project, Bat 1K^45^, Bird 10K^46^ and DNA Zoo. As the number of genomes grows, so too will the usefulness of comparative genomics in disease research and the development of therapeutic strategies. Preserving, rather than merely recording, the biodiversity of the Earth must be a priority. Through global scientific collaborations, and by making genomic resources available and accessible to all research communities, we can ensure that the legacy of genomics is not a digital archive of lost species.

## Methods

The number of samples (species) required to detect evolutionary conservation at a single base was estimated by applying a Poisson model of the distribution of substitution counts in the genome.

### Species selection, sample shipping and regulatory approvals

Species were selected to maximize branch length across the eutherian mammal phylogeny, and to capture genomes of species from previously unrepresented eutherian families. Of 172 species initially selected for inclusion, we obtained sufficiently high-quality DNA samples for genome sequencing for 137. DNA samples from collaborating institutions were shipped to the Broad Institute (*n* = 69) or Uppsala University (*n* = 68). For samples received at the Broad Institute that were then sent to Uppsala, shipping approval was secured from the US Fish and Wildlife Service. Institutional Animal Care and Use Committee approval was not required.

### Sample quality control, library construction and sequencing

DNA integrity for each sample was visualized via agarose gel (at the Broad Institute) or Agilent tape station (at Uppsala University). Samples passed quality control if the bulk of DNA fragments were greater than 5 kb. DNA concentration was then determined using Invitrogen Qubit dsDNA HS assay kit. For each of the samples that passed quality control, 1–3 μg of DNA was fragmented on the Covaris E220 Instrument using the 400-bp standard programme (10% duty cycle, 140 PIP, 200 cycles per burst, 55 s). Fragmented samples underwent SPRI double-size selection (0.55×, 0.7 × *f*) followed by PCR-free Illumina library construction following the manufacturer’s instructions (Kapa no. KK8232) using PCR-free adapters from Illumina (no. FC-121-3001). Final library fragment size distribution was determined on Agilent 2100 Bioanalyzer with High Sensitivity DNA Chips. Paired-end libraries were pooled, and then sequenced on a single lane of the Illumina HiSeq2500, set for Version 2 chemistry and 2×250-bp reads. This yielded a total of mean 375 million (s.d. = 125 million) reads per sample.

### Assembly and validation

For each species, we applied DISCOVAR de novo^11^ (discovardenovo-52488) (ftp://ftp.broadinstitute.org/pub/crd/DiscovarDeNovo/) to assemble the 2×250-bp read group, using the following command: DiscovarDeNovo READS = [READFILE] OUT_DIR = [SPECIES_ID]//[SPECIES_ID].discovar_files NUM_THREADS = 24 MAX_MEM_GB = 200G.

Coverage for each genome was automatically calculated by DISCOVAR, with a mean coverage of 40.1× (s.d.± 14×). We assessed genome assembly, gene set and transcriptome completeness using Benchmarking Universal Single-Copy Orthologs (BUSCO), which provides quantitative measures on the basis of gene content from near-universal single-copy orthologues^50^. BUSCO was run with default parameters, using the mammalian gene model set (mammalia_odb9, *n* = 4,104), using the following command: python ./BUSCO.py -i [input fasta] -o [output_file] -l ./mammalia_odb9/ -m genome -c 1 -sp. human.

Median contig N50 for existing RefSeq assemblies was calculated using the assembly statistics for the most recent release of 118 eutherian mammals with RefSeq assembly accession numbers. Assemblies were all classified as either reference genome or representative genome. Assembly statistics were downloaded from the NCBI on 10 April 2019.

#### Genome upgrades

We selected genomes from each eutherian order without a pre-existing long-contiguity assembly on the basis of (1) whether the underlying assembly met the minimum quality threshold needed for HiRise upgrades; and (2) whether a second sample of sufficient quality could be obtained from that individual. All upgrades were done with Dovetail Chicago libraries and assembled with HiRise 2.1, as previously described^51^.

### Estimating heterozygosity

#### Selection of assemblies for heterozygosity analysis

Heterozygosity statistics were calculated for all but four of our short read assemblies (*n* = 126) as well as eight Dovetail-upgraded genomes. Four failed because they were either too fragmented to analyse (*n* = 3) or because of undetermined errors (*n* = 1). One assembly was excluded because it was a second individual from a species that was already represented.

#### Heterozygosity calls

We applied the standard GATK pipeline with genotype quality banding to identify the callable fraction of the genome^52,53^. First, we used samtools to subsample paired reads from the unmapped .bam files^54^. After removing adaptor sequences from the selected reads, we used BWA-MEM to map reads to the reference genome scaffolds of >10 kb, removing duplicates using the PicardTools MarkDuplicates utility^55^. We then called heterozygous sites using standard GATK-Haplotypecaller specifications, and with additional gVCF banding at 0, 10, 20, 30, 40, 50 and 99 qualities. We used the fraction of the genome with genotype quality >15 for subsequent analyses. For the lists of high-confidence variant sites, we include only heterozygous positions after filtering at GQ >20, maximum DP <100, minimum DP >6, as described in the README file at http://broad.io/variants.

#### Inferring overall heterozygosity

To avoid confounding by sex chromosomes or complex regions, we excluded all scaffolds with less than 0.5 or greater than 2× of the average sample read depth, then calculated global heterozygosity as the fraction of heterozygous calls over the whole callable genome.

#### Calling SoH

We estimated the proportion of the genome within SoH using a metric designed for genomes with scaffold N50 shorter than the expected maximum length of runs of homozygosity (our median scaffold N50 is 62 kb). We first split all scaffolds into windows with a maximum length of 50 kb, with windows ranging from 20 to 50 kb for scaffolds <50 kb. For each window, we calculated the average number of heterozygous sites per bp. We discriminated windows with extremely low heterozygosity by using the Python 3.5.2 pomegranate package to fit a two-component Gaussian mixture model to the joint distribution of window heterozygosity, forcing the first component to be centred around the lower tail of the distribution and allowing the second to freely capture all the remaining heterozygosity variability^56,57^. As heterozygosity cannot be negative, and normal distributions near zero can cross into negative values, we used the normal cumulative distribution function to correct the posterior distribution by the negative excess—effectively fitting a truncated normal to the first component. The final SoH value was calculated using the posterior maximum likelihood classification between both components. We saw no significant correlation between contig N50 and SoH (Pearson correlation = 0.055, *P* = 0.57, *n* = 112).

#### Assessing the effect of the percentage of callable genome

We assessed whether the percentage of the genome that was callable (Supplementary Table 3) was likely to affect our analysis. The callable percentage was correlated with heterozygosity (*r* = −0.80, *P* < 2.2 × 10^−16^, *n* = 130), and weakly with SoH values (*r* = 0.18, *P* = 0.06, *n* = 112). There is no significant difference in callable percentage among IUCN categories (analysis of variance *P* = 0.98, *n* = 122) or between captive and wild populations (*t*-test *P* = 0.81, *n* = 120).

#### Analysing patterns of diversity

We excluded two genomes with exceptionally high heterozygosity (heterozygosity >0.02; >5 s.d. above the mean). Both were of non-endangered species, and thus removing them made our determination of lower heterozygosity in endangered species more conservative. Of the remaining 124 genomes, we excluded 19 with allelic balance values that were more than one s.d. above the mean (>0.36). Abnormally high allelic balance can indicate sequencing biases with potential for artefacts in estimates of heterozygosity and/or SoH. Our final dataset contains heterozygosity values for 105 genomes and SoH values for 98 genomes (Supplementary Table 3). For seven genomes, we were unable to estimate SoH because the two components of the Gaussian mixture model overlapped completely. To ask about a possible directional relationship between level of IUCN concern and overall heterozygosity or SoH, we applied regression using the IUCN category as an ordinal predictor. We also asked about the relationship of diversity metrics to a set of species-level phenotypes for which correlations were previously reported (Extended Data Table 3).

### Alignment

The alignment was generated using the progressive mode of Cactus^37,58^. The topology used for the guide tree of the alignment was taken from TimeTree^47^; the branch lengths of the guide tree were generated by a least-squares fit from a distance matrix. The distance matrix was based on the UCSC 100-way phyloP fourfold-degenerate site tree^38^ for those species that had corresponding entries in the 100-way tree. For species not present in the 100-way tree, distance matrix entries were more coarsely estimated using the distance estimated from Mash^59^ to the closest relative included in the 100-way data.

Cactus does not attempt to fully resolve the gene tree when multiple duplications take place along a single branch, as there is an implicit restriction in Cactus that a duplication event be represented as multiple regions in the child species aligned to a single region in the parent species. This precludes representing discordance between the gene tree and species tree that could occur with incomplete lineage-sorting or horizontal transfer. However, the guide tree has a minimal effect on the alignment, with little difference between alignments with different trees—even when using a tree that is purposely wrong^37^. Phenomena such as incomplete lineage sorting that affect a subset of species are unlikely to substantially affect the detection of purifying selection across the whole eutherian lineage described in Fig. 3.

The alignment was generated in several steps, on account of its large scale. First, a backbone alignment of several long contiguity assemblies was generated, using the genomes of two non-placental mammals (Tasmanian devil (*Sarcophilus harrisii*) and platypus (*Ornithorhynchus anatinus*)), to inform the reconstruction of the placental root. Next, separate clade alignments were generated for each major clade in the alignment: Euarchonta, Glires, Laurasiatheria, Afrotheria and Xenarthra. The roots of these clade alignments were then aligned to the corresponding ancestral genomes from the backbone, stitching these alignments together to create the final alignment. The process of aligning a genome to an existing ancestor is complex and further described in an accompanying Article that introduces the progressive mode of Cactus^37^.

We created a neutral model for the conservation analysis using ancestral repeats detected by RepeatMasker^60^ on the eutherian ancestral genome produced in the Cactus alignment (tRNA and low-complexity repeats were removed). To fit the neutral model, we used phyloFit from the PHAST^61^ package, using the REV (generalized reversible) model and EM optimization method. The training input was a MAF exported on columns from the set of ancestral repeats mentioned above. Because phyloFit does not support alignment columns that contain duplicates, if a genome had more than one sequence in a single alignment block, these were replaced with a single entry representing the consensus base at each column.

We extracted initial conservation scores using phyloP from the PHAST^61^ package on a MAF exported using human as a reference. We converted the phyloP scores (which represent log-scaled *P* values of acceleration or conservation) into *P* values, then into *q* values using the FDR-correction of Benjamini and Hochberg^62^. Any column with a resulting *q* value less than 0.05 was deemed significantly conserved or accelerated.

The alignment—as well as conservation annotations—are available at https://cglgenomics.ucsc.edu/data/cactus/.

### Reporting summary

Further information on research design is available in the Nature Research Reporting Summary linked to this paper.

## Online content

Any methods, additional references, Nature Research reporting summaries, source data, extended data, supplementary information, acknowledgements, peer review information; details of author contributions and competing interests; and statements of data and code availability are available at 10.1038/s41586-020-2876-6.

### Supplementary information

Supplementary Tables This file contains Supplementary Tables 1-3.

Reporting Summary

### Source data

Source Data Fig. 2

## Figures and Tables

**Extended Data Table 1:**
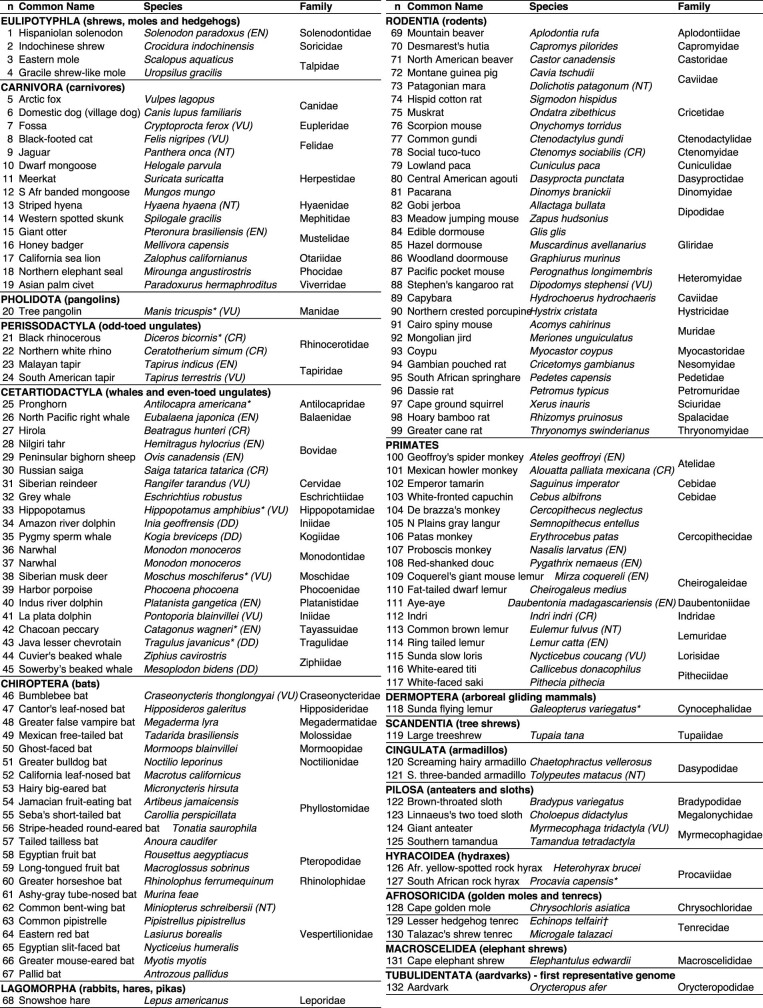
The Zoonomia Project data includes 132 genome assemblies

These assemblies include 131 different species, with 2 narwhals (male and female), and 10 genomes upgraded to longer contiguity (including upgrade of an existing assembly for *E. telfairi*).

Species of concern on the IUCN Red List are indicated as near-threatened (NT), vulnerable (V), endangered (EN) or critically endangered (CR).

*Upgraded to longer contiguity.

†Upgraded to longer contiguity using existing assembly.

**Extended Data Table 2:**
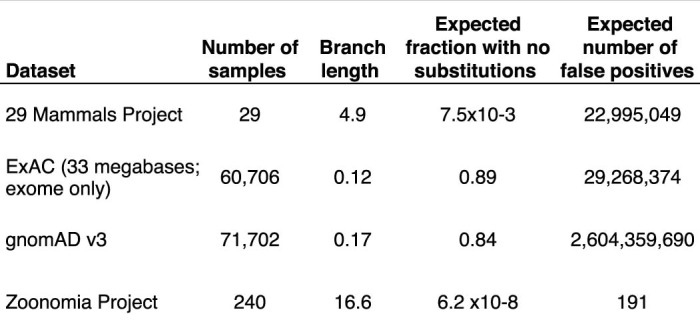
Power to detect constraint across datasets

The expected number of variants conserved by chance (false positives) was estimated for four genomic resources (the 29 Mammals Project^7^ dataset, the human-only ExAC^14^ and gnomAD v.3^65^ datasets, and the Zoonomia Project dataset) by applying a Poisson model of the distribution of substitution counts in the genome. Branch length for gnomAD was estimated by dividing 526,001,545 single-nucleotide variants by 3.088 gigabases (size of the human genome). Branch length for Zoonomia was measured as the number of substitutions per site in the phyloP analysis of the Cactus alignment.

**Extended Data Table 3:**
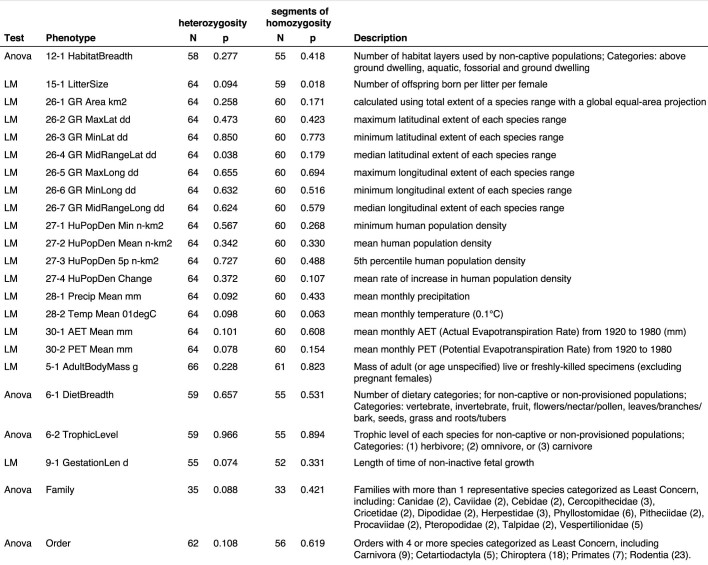
Diversity statistics are not correlated with other species-level phenotypes

All phenotypes in the PanTHERIA database^30^ for which at least 75% of the 75 species of least concern had a value were included in the analysis. For continuous phenotypes, values were standardized to *Z*-scores before analysis (latitude was calculated as an absolute value) and correlation measured by fitting a linear model using the core R function lm. For categorical phenotypes with more than two categories, group means were compared using the core R function aov to fit an analysis of variance model. None was significant after Bonferroni correction for the number of traits considered (21).

## Data Availability

The project website is http://zoonomiaproject.org/. Details of each Zoonomia genome assembly—including NCBI GenBank^63^ accession numbers—are provided in Supplementary Table 1. Sequence data and genome assemblies are available at https://www.ncbi.nlm.nih.gov/. Variant lists for each species are provided at http://broad.io/variants. Further source data for Fig. 2 are provided in the Zoonomia GitHub repository (10.5281/zenodo.3887432). The Cactus alignment is provided at https://cglgenomics.ucsc.edu/data/cactus/. A visualization of the alignments and phyloP data is available by loading our assembly hub into the UCSC browser^64^ by copying the hub link https://comparative-genomics-hubs.s3-us-west-2.amazonaws.com/200m_hub.txt into the Track Hubs page. There are no restrictions on use. Source data are provided with this paper.

## Code availability

The DISCOVAR de novo assembly code is available at https://github.com/broadinstitute/discovar_de_novo/releases/tag/v52488 (10.5281/zenodo.3870889), the Cactus pipeline is available at https://github.com/ComparativeGenomicsToolkit/cactus (10.5281/zenodo.3873410) and code for other analyses is available at https://github.com/broadinstitute/Zoonomia/ (10.5281/zenodo.3887432).
