# Supplementary material for: A comparative genomics multitool for scientific discovery and conservation
Source: Nature. 2020 Nov 11;587(7833):240–5. doi: 10.1038/s41586-020-2876-6 (PMC7759459; doi:10.1038/s41586-020-2876-6)
Supplement: Supplementary file 1 — This file contains Supplementary Tables 1-3. [file 41586_2020_2876_MOESM1_ESM.pdf]

natureresearch
https://doi.org/10.1038/s41586-020-2876-6

Supplementary information

A comparative genomics multitool for scientific discovery and conservation

In the format provided by the authors and unedited

SI Guide

The Supplementary Information files for “A comparative genomics multitool for scientific discovery and conservation” consist of the following:

Supplemental Table 1. Genome assemblies made for the Zoonomia Project
Supplemental Table 2. Genomes in Cactus alignment
Supplemental Table 3. Genetic diversity in Zoonomia genome assemblies

Supplementary Table 1. Genome assembly statistics for all attemped and successful Zoonomia Project genomes.

Of 173 species and subspecies initially planned for inclusion in the Zoonomia Project, genome assemblies have been generated and released for 132. For the remainder, acquisition of high quality samples was a major impediment. Set A: Discovar only genomes; Set B: Genomes upgraded to longer contiguity; Set C: Assembly failed; Set D: Sample failed QC; Set E: Sample not found; ** Genome assembly statistics output by DISCOVAR *de novo*.

All samples were collected and shipped in compliance with the applicable regulations for sample collection, transfer, export and import.

Set	Species	Common Name	Order	Family	IUCN	Biosample	Provider Institution	Provider contact	Sequencing location	Genbank accession	Contig N50 (bp) **	Scaffold N50 (bp)**	Size (Gb; contigs >1kb)**	Mean base quality**	Coverage**	Busco Complete (n=4104)	BUSCO Single Copy	BUSCO Duplicated	BUSCO Fragmented	BUSCO Missing
A	*Solenodon paradoxus*	Hispaniolan solenodon	Eulipotyphla	Solenodontidae	Endangered	SAMN07678062	Liverpool School of Tropical Medicine	Nicholas Casewell	Uppsala University	GCA_004363575.1	244,273	422,311	2.05	35.8	29.3	90.4%	90.1%	0.3%	6.9%	2.7%
A	*Crocidura indochinensis*	Indochinese shrew	Eulipotyphla	Soricidae	Least Concern	SAMN07678044	University of Manitoba	Kevin Campbell	Uppsala University	GCA_004027635.1	8,485	8,747	2.23	33.4	32.2	20.6%	20.1%	0.5%	36.5%	42.9%
A	*Scalopus aquaticus*	Eastern mole	Eulipotyphla	Talpidae	Least Concern	SAMN07678045	University of Manitoba	Kevin Campbell	Uppsala University	GCA_004024925.1	80,361	105,245	1.88	33.1	46.1	82.3%	81.9%	0.4%	13.0%	4.7%
A	*Uropsilus gracilis*	Gracile shrew-like mole	Eulipotyphla	Talpidae	Least Concern	SAMN07678046	University of Manitoba	Kevin Campbell	Uppsala University	GCA_004024945.1	50,461	64,331	1.88	33.9	54.2	65.1%	64.8%	0.3%	24.8%	10.1%
A	*Vulpes lagopus*	Arctic fox	Carnivora	Canidae	Least Concern	SAMN07678118	San Diego Zoo	Oliver Ryder	Uppsala University	GCA_004023825.1	88,551	123,667	2.32	35.8	36.8	81.5%	80.3%	1.2%	13.5%	5.0%
A	*Canis lupus familiaris*	Domestic dog (village dog)	Carnivora	Canidae	Least Concern	SAMN07678141	Cornell University	Adam Boyko	Broad Institute	GCA_004027395.1	110,028	173,941	2.34	33.6	66.5	85.0%	84.0%	1.0%	10.3%	4.7%
A	*Cryptoprocta ferox*	Fossa	Carnivora	Eupleridae	Vulnerable	SAMN07678074	San Diego Zoo	Oliver Ryder	Broad Institute	GCA_004023885.1	137,953	186,904	2.32	32.2	46.3	85.9%	84.9%	1.0%	10.6%	3.5%
A	*Felis nigripes*	Black-footed cat	Carnivora	Felidae	Vulnerable	SAMN07678084	San Diego Zoo	Oliver Ryder	Broad Institute	GCA_004023925.1	16,930	19,727	2.27	33.5	17.1	37.7%	37.5%	0.2%	40.8%	21.5%
A	*Panthera onca*	Jaguar	Carnivora	Felidae	Near threatened	SAMN07678103	San Diego Zoo	Oliver Ryder	Broad Institute	GCA_004023805.1	66,276	123,177	2.37	33.2	60.7	78.4%	78.0%	0.4%	15.7%	5.9%
A	*Helogale parvula*	Dwarf mongoose	Carnivora	Herpestidae	Least Concern	SAMN07678086	San Diego Zoo	Oliver Ryder	Uppsala University	GCA_004023845.1	116,675	184,757	2.33	34.0	32.3	83.9%	83.2%	0.7%	11.8%	4.3%
A	*Suricata suricatta*	Meerkat	Carnivora	Herpestidae	Least Concern	SAMN07678040	Zoo New England	Eric Baitchman	Broad Institute	GCA_004023905.1	156,832	196,874	2.36	34.1	46.9	86.1%	85.0%	1.1%	10.0%	3.9%
A	*Mungos mungo*	South African banded mongoose	Carnivora	Herpestidae	Least Concern	SAMN07678097	San Diego Zoo	Oliver Ryder	Uppsala University	GCA_004023785.1	189,084	247,492	2.35	35.9	46.7	87.4%	86.6%	0.8%	9.1%	3.5%
A	*Hyaena hyaena*	Striped hyena	Carnivora	Hyaenidae	Near threatened	SAMN07678047	Smithsonian Insitute	Klaus Koepfli	Broad Institute	GCA_004023945.1	53,836	69,191	2.34	32.7	31.3	71.1%	70.1%	1.0%	20.0%	8.9%
A	*Spilogale gracilis*	Western spotted skunk	Carnivora	Mephitidae	Least Concern	SAMN07678050	Smithsonian Insitute	Klaus Koepfli	Uppsala University	GCA_004023965.1	66,417	90,418	2.33	33.1	33.2	76.7%	76.1%	0.6%	17.1%	6.2%
A	*Pteronura brasiliensis*	Giant otter	Carnivora	Mustelidae	Endangered	SAMN07678049	Smithsonian Insitute	Klaus Koepfli	Broad Institute	GCA_004024605.1	99,882	130,554	2.41	32.6	47.7	82.0%	81.4%	0.6%	13.3%	4.7%
A	*Mellivora capensis*	Honey badger	Carnivora	Mustelidae	Least Concern	SAMN07678094	San Diego Zoo	Oliver Ryder	Broad Institute	GCA_004024625.1	66,331	82,519	2.63	31.8	38.8	73.0%	72.1%	0.9%	18.5%	8.5%
A	*Zalophus californianus*	California sea lion	Carnivora	Otariidae	Least Concern	SAMN07678053	North Carolina State University	Matthew Breen	Broad Institute	GCA_004024565.1	97,878	143,278	2.35	33.3	89.2	85.0%	83.3%	1.7%	10.3%	4.7%
A	*Mirounga angustirostris*	Northern elephant seal	Carnivora	Phocidae	Least Concern	SAMN07678016	MGH/HMS	Allyson Hindle	Broad Institute	GCA_004023865.1	54,778	67,070	2.31	32.3	35.6	72.3%	71.6%	0.7%	20.5%	7.2%
A	*Paradoxurus hermaphroditus*	Asian palm civet	Carnivora	Viverridae	Least Concern	SAMN07678048	Smithsonian Insitute	Klaus Koepfli	Broad Institute	GCA_004024585.1	67,775	77,417	2.32	33.1	36.9	74.8%	74.2%	0.6%	18.4%	6.8%
A	*Manis tricuspis*	Tree pangolin	Pholidota	Manidae	Vulnerable	SAMN07678093	San Diego Zoo	Oliver Ryder	Broad Institute	GCA_004765945.1	33,856	36,113	2.37	34.6	30.2	60.1%	59.5%	0.6%	27.2%	12.7%
A	*Diceros bicornis*	Black rhinocerous	Perissodactyla	Rhinocerotidae	Critically Endangered	SAMN07678078	San Diego Zoo	Oliver Ryder	Broad Institute	GCA_004027315.1	114,126	151,674	2.44	32.4	44.9	83.4%	82.9%	0.5%	12.9%	3.7%
A	*Ceratotherium simum cottoni*	Northern white rhino	Perissodactyla	Rhinocerotidae	Critically Endangered	SAMN07678069	San Diego Zoo	Oliver Ryder	Broad Institute	GCA_004027795.1	23,748	25,821	2.40	32.9	29.2	48.7%	47.1%	1.6%	33.3%	18.0%
A	*Tapirus indicus*	Malayan tapir	Perissodactyla	Tapiridae	Endangered	SAMN07678113	San Diego Zoo	Oliver Ryder	Broad Institute	GCA_004024905.1	234,383	320,013	2.33	34.4	41.4	84.1%	83.7%	0.4%	12.5%	3.4%
A	*Tapirus terrestris*	South American tapir	Perissodactyla	Tapiridae	Vulnerable	SAMN07678114	San Diego Zoo	Oliver Ryder	Broad Institute	GCA_004025025.1	184,442	207,300	2.34	44.0	44.2	82.0%	81.6%	0.4%	14.0%	4.0%
A	*Antilocapra americana*	Pronghorn	Cetartiodactyla	Antilocapridae	Least Concern	SAMN07678063	San Diego Zoo	Oliver Ryder	Broad Institute	GCA_004027515.1	75,180	90,528	2.41	32.5	49.3	80.4%	78.3%	2.1%	13.6%	6.0%
A	*Eubalaena japonica*	North Pacific right whale	Cetartiodactyla	Balaenidae	Endangered	SAMN07678124	Southwest Fisheries Science Center	Phillip Morin	Broad Institute	GCA_004363455.1	41,990	48,127	2.31	33.7	40.0	69.0%	67.7%	1.3%	22.6%	8.4%
A	*Beatragus hunteri*	Hirola	Cetartiodactyla	Bovidae	Critically Endangered	SAMN07678065	San Diego Zoo	Oliver Ryder	Broad Institute	GCA_004027495.1	60,436	72,932	2.55	34.6	23.1	76.6%	75.9%	0.7%	16.8%	6.6%
A	*Hemitragus hylocrius*	Nilgiri tahr	Cetartiodactyla	Bovidae	Endangered	SAMN07678087	San Diego Zoo	Oliver Ryder	Broad Institute	GCA_004026825.1	69,919	89,641	2.58	32.5	29.4	79.9%	78.4%	1.5%	14.1%	6.0%
A	*Ovis canadensis cremnobates*	Peninsular bighorn sheep	Cetartiodactyla	Bovidae	Endangered	SAMN07678102	San Diego Zoo	Oliver Ryder	Broad Institute	GCA_004026945.1	62,884	78,524	2.57	33.9	25.2	76.3%	75.2%	1.1%	17.0%	6.7%
A	*Saiga tatarica tatarica*	Russian saiga	Cetartiodactyla	Bovidae	Critically Endangered	SAMN07678151	San Diego Zoo	Oliver Ryder	Broad Institute	GCA_004024985.1	7,893	7,998	2.34	33.1	18.8	23.8%	23.2%	0.6%	42.4%	33.8%
A	*Rangifer tarandus*	Siberian reindeer	Cetartiodactyla	Cervidae	Vulnerable	SAMN07678109	San Diego Zoo	Oliver Ryder	Uppsala University	GCA_004026565.1	91,907	106,338	2.50	36.3	45.9	80.1%	77.8%	2.3%	13.1%	6.8%
A	*Eschrichtius robustus*	Grey whale	Cetartiodactyla	Eschrichtiidae	Least Concern	SAMN07678123	Southwest Fisheries Science Center	Phillip Morin	Broad Institute	GCA_004363415.1	78,871	107,875	2.33	32.3	40.5	79.2%	78.1%	1.1%	15.2%	5.6%
A	*Hippopotamus amphibius*	Hippopotamus	Cetartiodactyla	Hippopotamidae	Vulnerable	SAMN07678089	San Diego Zoo	Oliver Ryder	Broad Institute	GCA_004027065.1	84,845	98,945	2.39	33.9	77.0	80.8%	79.0%	1.8%	13.7%	5.5%
A	*Inia geoffrensis*	Amazon river dolphin	Cetartiodactyla	Iniidae	Data deficient	SAMN07678125	Southwest Fisheries Science Center	Phillip Morin	Broad Institute	GCA_004363515.1	30,246	33,256	2.26	31.8	42.5	58.7%	57.8%	0.9%	25.3%	16.0%
A	*Kogia breviceps*	Pygmy sperm whale	Cetartiodactyla	Kogiidae	Data deficient	SAMN07678126	Southwest Fisheries Science Center	Phillip Morin	Broad Institute	GCA_004363705.1	31,609	34,952	2.37	34.5	38.8	58.8%	58.1%	0.7%	27.3%	13.9%
A	*Monodon monoceros*	Narwhal	Cetartiodactyla	Monodontidae	Least Concern	SAMN07678023	Ocean Genome Legacy/Northeastern University	Dan Distel	Broad Institute	GCA_004027045.1	78,802	99,265	2.31	33.2	34.7	76.1%	75.1%	1.0%	17.5%	6.4%
A	*Monodon monoceros (male)*	Narwhal	Cetartiodactyla	Monodontidae	Least Concern	SAMN07678024	Ocean Genome Legacy/Northeastern University	Dan Distel	Broad Institute	GCA_004026685.1	72,592	93,992	2.29	34.2	23.2	75.2%	74.2%	1.0%	17.7%	7.1%
A	*Moschus moschiferus*	Siberian musk deer	Cetartiodactyla	Moschidae	Vulnerable	SAMN07678096	San Diego Zoo	Oliver Ryder	Broad Institute	GCA_004024705.1	39,944	54,975	2.62	34.1	64.9	73.2%	71.6%	1.6%	18.7%	8.1%
A	*Phocoena phocoena*	Harbor porpoise	Cetartiodactyla	Phocoenidae	Least Concern	SAMN07678060	The Museum of Vertebrate Zoology at Berkeley	Michael Nachman	Broad Institute	GCA_004363495.1	105,655	138,932	2.32	34.5	56.4	80.9%	79.8%	1.1%	13.0%	6.1%
A	*Platanista gangetica minor*	Indus river dolphin	Cetartiodactyla	Platanistidae	Endangered	SAMN07678149	Southwest Fisheries Science Center	Phillip Morin	Broad Institute	GCA_004363435.1	23,592	27,013	2.36	34.1	28.0	49.7%	48.5%	1.2%	34.6%	15.7%

Supplementary Table 1. Genome assembly statistics for all attemped and successful Zoonomia Project genomes.

Of 173 species and subspecies initially planned for inclusion in the Zoonomia Project, genome assemblies have been generated and released for 132. For the remainder, acquisition of high quality samples was a major impediment. Set A: Discovar only genomes; Set B: Genomes upgraded to longer contiguity; Set C: Assembly failed; Set D: Sample failed QC; Set E: Sample not found; ** Genome assembly statistics output by DISCOVAR *de novo*.

All samples were collected and shipped in compliance with the applicable regulations for sample collection, transfer, export and import.

Set	Species	Common Name	Order	Family	IUCN	Biosample	Provider Institution	Provider contact	Sequencing location	Genbank accession	Contig N50 (bp) **	Scaffold N50 (bp)**	Size (Gb; contigs >1kb)**	Mean base quality**	Coverage**	Busco Complete (n=4104)	BUSCO Single Copy	BUSCO Duplicated	BUSCO Fragmented	BUSCO Missing
A	*Pontoporia blainvillei*	La plata dolphin	Cetartiodactyla	Iniidae	Vulnerable	SAMN07678127	Southwest Fisheries Science Center	Phillip Morin	Broad Institute	GCA_004363935.1	4,149	4,148	1.17	32.8	55.3	23.3%	22.9%	0.4%	24.9%	51.8%
A	*Catagonus wagneri*	Chacoan peccary	Cetartiodactyla	Tayassuidae	Endangered	SAMN07678067	San Diego Zoo	Oliver Ryder	Broad Institute	GCA_004024745.1	173,559	236,870	2.12	33.6	87.1	85.8%	85.0%	0.8%	10.0%	4.2%
A	*Tragulus javanicus*	Java lesser chevrotain	Cetartiodactyla	Tragulidae	Data deficient	SAMN07678116	San Diego Zoo	Oliver Ryder	Uppsala University	GCA_004024965.1	92,271	101,971	2.25	35.9	43.0	76.9%	75.2%	1.7%	16.1%	7.0%
A	*Ziphius cavirostris*	Cuvier's beaked whale	Cetartiodactyla	Ziphiidae	Least Concern	SAMN07678128	Southwest Fisheries Science Center	Phillip Morin	Broad Institute	GCA_004364475.1	8,393	8,552	1.97	34.6	30.6	29.5%	29.0%	0.5%	37.8%	32.7%
A	*Mesoplodon bidens*	Sowerby's beaked whale	Cetartiodactyla	Ziphiidae	Data deficient	SAMN07678145	Southwest Fisheries Science Center	Phillip Morin	Uppsala University	GCA_004027085.1	37,451	44,011	2.25	34.9	32.4	63.1%	61.6%	1.5%	24.3%	12.6%
A	*Craseonycteris thonglongyai*	Bumblebee bat	Chiroptera	Craseonycteridae	Vulnerable	SAMN07678039	University College Dublin	Emma Teeling	Uppsala University	GCA_004027555.1	28,297	31,618	1.93	35.1	52.2	60.7%	60.0%	0.7%	26.2%	13.1%
A	*Hipposideros galeritus*	Cantor's leaf-nosed bat	Chiroptera	Hipposideridae	Least Concern	SAMN07678026	Texas Tech University	David Ray	Uppsala University	GCA_004027415.1	38,797	44,060	2.16	36.1	46.0	65.7%	62.9%	2.8%	21.3%	13.0%
A	*Megaderma lyra*	Greater false vampire bat	Chiroptera	Megadermatidae	Least Concern	SAMN07678029	Texas Tech University	David Ray	Uppsala University	GCA_004026885.1	108,673	147,888	2.03	35.9	49.8	87.6%	87.0%	0.6%	8.2%	4.2%
A	*Tadarida brasiliensis*	Mexican free-tailed bat	Chiroptera	Molossidae	Least Concern	SAMN07678131	Texas Tech University	Robert Baker	Uppsala University	GCA_004025005.1	27,768	30,088	2.36	35.2	30.3	60.0%	58.9%	1.1%	25.7%	14.3%
A	*Mormoops blainvillei*	Ghost-faced bat	Chiroptera	Mormoopidae	Least Concern	SAMN07678133	Queen Mary University	Stephen Rossiter	Uppsala University	GCA_004026545.1	147599	161296	2.04	36	33	85.2%	84.6%	6.0%	10.1%	4.7%
A	*Noctilio leporinus*	Greater bulldog bat	Chiroptera	Noctilionidae	Least Concern	SAMN07678134	Queen Mary University	Stephen Rossiter	Uppsala University	GCA_004026585.1	143,138	202,442	2.01	35.0	42.9	84.9%	83.7%	1.2%	10.0%	5.1%
A	*Macrotus californicus*	California leaf-nosed bat	Chiroptera	Phyllostomidae	Least Concern	SAMN07678130	Texas Tech University	Robert Baker	Uppsala University	GCA_007922815.1	22,729	23,701	1.84	33.1	47.7	60.2%	59.4%	0.8%	21.8%	12.0%
A	*Micronycteris hirsuta*	Hairy big-eared bat	Chiroptera	Phyllostomidae	Least Concern	SAMN07678146	Texas Tech University	David Ray	Uppsala University	GCA_004026765.1	68,746	75,954	2.14	35.7	39.4	75.7%	75.1%	0.6%	17.1%	7.2%
A	*Artibeus jamaicensis*	Jamacian fruit-eating bat	Chiroptera	Phyllostomidae	Least Concern	SAMN07678132	Queen Mary University	Stephen Rossiter	Uppsala University	GCA_004027435.1	37,407	37,407	2.17	35.5	30.2	66.4%	65.8%	0.6%	22.1%	11.5%
A	*Carollia perspicillata*	Seba's short-tailed bat	Chiroptera	Phyllostomidae	Least Concern	SAMN07678055	The Museum of Vertebrate Zoology at Berkeley	Michael Nachman	Uppsala University	GCA_004027735.1	17,377	18,416	2.01	33.5	27.7	43.5%	43.3%	0.2%	33.7%	22.8%
A	*Tonatia saurophila*	Stripe-headed round-eared bat	Chiroptera	Phyllostomidae	Least Concern	SAMN07678031	Texas Tech University	David Ray	Uppsala University	GCA_004024845.1	148,656	173,079	2.02	34.9	46.1	83.6%	83.2%	0.4%	11.2%	5.2%
A	*Anoura caudifer*	Tailed tailless bat	Chiroptera	Phyllostomidae	Least Concern	SAMN07678033	Texas Tech University	David Ray	Uppsala University	GCA_004027475.1	150,858	196,885	2.10	34.4	52.5	86.0%	85.6%	0.4%	9.5%	4.5%
A	*Rousettus aegyptiacus*	Egyptian fruit bat	Chiroptera	Pteropodidae	Least Concern	SAMN07678121	Charles University, Prague	Pavel Hulva	Uppsala University	GCA_004024865.1	104,971	131,538	1.90	33.6	35.7	85.8%	85.3%	0.5%	10.4%	3.8%
A	*Macroglossus sobrinus*	Long-tongued fruit bat	Chiroptera	Pteropodidae	Least Concern	SAMN07678028	Texas Tech University	David Ray	Uppsala University	GCA_004027375.1	351,412	471,405	1.85	35.5	52.7	90.5%	90.3%	0.2%	6.2%	3.3%
A	*Rhinolophus ferrumequinum*	Greater horseshoe bat	Chiroptera	Rhinolophidae	Least Concern	SAMN07678122	Charles University, Prague	Pavel Hulva	Uppsala University	GCA_007922735.1	134,856	165,950	2.08	34.4	39.7	87.7%	86.8%	0.9%	8.3%	4.0%
A	*Murina feae*	Ashy-gray tube-nosed bat	Chiroptera	Vespertilionidae	Least Concern	SAMN07678148	Museum Geneve	Manuel Ruedi	Uppsala University	GCA_004026665.1	26,934	30,093	2.06	35.4	33.3	57.0%	56.3%	0.7%	28.9%	14.1%
A	*Miniopterus schreibersii*	Common bent-wing bat	Chiroptera	Vespertilionidae	Near threatened	SAMN07678147	Museum Geneve	Manuel Ruedi	Uppsala University	GCA_004026525.1	85,056	111,584	1.72	35.0	24.8	85.5%	84.9%	0.6%	10.9%	3.6%
A	*Pipistrellus pipistrellus*	Common pipistrelle	Chiroptera	Vespertilionidae	Least Concern	SAMN07678120	Charles University, Prague	Pavel Hulva	Uppsala University	GCA_004026625.1	30,524	39,435	1.87	35.2	52.8	60.1%	58.9%	1.2%	26.8%	13.1%
A	*Lasiurus borealis*	Eastern red bat	Chiroptera	Vespertilionidae	Least Concern	SAMN07678027	Texas Tech University	David Ray	Uppsala University	GCA_004026805.1	35,447	41,005	2.71	35.2	42.1	62.3%	49.9%	1.2%	25.5%	12.2%
A	*Nycticeius humeralis*	Egyptian slit-faced bat	Chiroptera	Vespertilionidae	Least Concern	SAMN07678030	Texas Tech University	David Ray	Uppsala University	GCA_007922795.1	20,388	21,762	2.24	34.3	41.7	46.3%	44.9%	1.4%	34.3%	19.4%
A	*Myotis myotis*	Greater mouse-eared bat	Chiroptera	Vespertilionidae	Least Concern	SAMN07678038	University College Dublin	Emma Teeling	Uppsala University	GCA_004026985.1	23,271	26,512	1.96	35.4	47.8	54.4%	53.2%	1.2%	30.0%	15.6%
A	*Antrozous pallidus*	Pallid bat	Chiroptera	Vespertilionidae	Least Concern	SAMN07678034	Texas Tech University	David Ray	Uppsala University	GCA_007922775.1	58,793	81,996	2.64	34.6	35.7	76.8%	76.0%	0.8%	16.2%	7.0%
A	*Lepus Americanus*	Snowshoe hare	Lagomorpha	Leporidae	Least Concern	SAMN07678042	University of Montana	Jeffrey Good	Broad Institute	GCA_004026855.1	19,314	21,687	2.59	32.5	32.6	45.6%	44.6%	1.0%	32.7%	21.7%
A	*Aplodontia rufa*	Mountain beaver	Rodentia	Aplodontiidae	Least Concern	SAMN07678037	Texas A&M	Bill Murphy	Broad Institute	GCA_004027875.1	40,591	46,219	2.59	33	30.6	67.6%	66.8%	0.8%	21.2%	11.2%
A	*Capromys pilorides*	Desmarest's hutia	Rodentia	Capromyidae	Least Concern	SAMN07678066	San Diego Zoo	Oliver Ryder	Uppsala University	GCA_004027915.1	6,891	6,952	2.17	34.7	26.1	33.6%	33.1%	0.5%	29.1%	37.3%
A	*Castor canadensis*	North American beaver	Rodentia	Castoridae	Least Concern	SAMN07678136	Harvard Medical School	Vadim Gladyshev	Uppsala University	GCA_004027675.1	55,022	62,169	2.51	36.2	30.4	72.6%	71.8%	0.8%	19.2%	8.2%
A	*Cavia tschudii*	Montane guinea pig	Rodentia	Caviidae	Least Concern	SAMN07678017	Texas A&M	Bill Murphy	Uppsala University	GCA_004027695.1	79,889	113,293	2.63	34.7	34.8	82.8%	82.4%	0.4%	11.6%	5.6%
A	*Dolichotis patagonum*	Patagonian mara	Rodentia	Caviidae	Near threatened	SAMN07678081	San Diego Zoo	Oliver Ryder	Uppsala University	GCA_004027295.1	40,160	45,179	2.66	34.4	30.8	70.6%	70.2%	0.4%	19.0%	10.4%
A	*Sigmodon hispidus*	Hispid cotton rat	Rodentia	Cricetidae	Least Concern	SAMN07678138	Harvard Medical School	Vadim Gladyshev	Uppsala University	GCA_004025045.1	76,554	114,306	2.46	35.9	43.2	82.7%	80.5%	2.2%	11.8%	5.5%
A	*Ondatra zibethicus*	Muskrat	Rodentia	Cricetidae	Least Concern	SAMN07678059	The Museum of Vertebrate Zoology at Berkeley	Michael Nachman	Uppsala University	GCA_004026605.1	79,948	96,834	2.39	36.0	43.9	80.7%	79.7%	1.0%	14.0%	5.3%
A	*Onychomys torridus*	Scorpion mouse	Rodentia	Cricetidae	Least Concern	SAMN07678022	Cornell University	Bret Pasch	Uppsala University	GCA_004026725.1	26,822	31,336	2.46	34.9	46.8	58.9%	58.3%	0.6%	28.0%	13.1%
A	*Ctenodactylus gundi*	Common gundi	Rodentia	Ctenodactylidae	Least Concern	SAMN07678018	Texas A&M	Bill Murphy	Uppsala University	GCA_004027205.1	246,904	408,695	2.07	35.8	51.0	90.1%	89.7%	0.4%	6.5%	3.4%
A	*Ctenomys sociabilis*	Social tuco-tuco	Rodentia	Ctenomyidae	Critically Endangered	SAMN07678051	University of New Hampshire	Matt MacManes	Broad Institute	GCA_004027165.1	41,379	54,015	2.60	32.9	26.5	65.5%	63.3%	2.2%	21.6%	12.9%
A	*Cuniculus paca*	Lowland paca	Rodentia	Cuniculidae	Least Concern	SAMN07678075	San Diego Zoo	Oliver Ryder	Broad Institute	GCA_004365215.1	9,059	9,234	2.17	33.5	23.4	26.0%	25.8%	0.2%	38.2%	35.8%
A	*Dasyprocta punctata*	Central American agouti	Rodentia	Dasyproctidae	Least Concern	SAMN07678076	San Diego Zoo	Oliver Ryder	Broad Institute	GCA_004363535.1	44,076	50,437	2.72	32.1	40.0	67.0%	66.4%	0.6%	20.9%	12.1%
A	*Dinomys branickii*	Pacarana	Rodentia	Dinomyidae	Least Concern	SAMN07678079	San Diego Zoo	Oliver Ryder	Uppsala University	GCA_004027595.1	75,870	91,033	2.37	36.3	31.9	77.3%	76.5%	0.8%	15.0%	7.7%
A	*Allactaga bullata*	Gobi jerboa	Rodentia	Dipodidae	Least Concern	SAMN07678035	University of California, San Diego	Kimberly Cooper	Uppsala University	GCA_004027895.1	37,276	44,428	2.67	35.1	29.1	54.5%	53.7%	0.8%	28.9%	16.6%

Supplementary Table 1. Genome assembly statistics for all attemped and successful Zoonomia Project genomes.

Of 173 species and subspecies initially planned for inclusion in the Zoonomia Project, genome assemblies have been generated and released for 132. For the remainder, acquisition of high quality samples was a major impediment. Set A: Discovar only genomes; Set B: Genomes upgraded to longer contiguity; Set C: Assembly failed; Set D: Sample failed QC; Set E: Sample not found; ** Genome assembly statistics output by DISCOVAR *de novo*.

All samples were collected and shipped in compliance with the applicable regulations for sample collection, transfer, export and import.

Set	Species	Common Name	Order	Family	IUCN	Biosample	Provider Institution	Provider contact	Sequencing location	Genbank accession	Contig N50 (bp) **	Scaffold N50 (bp)**	Size (Gb; contigs >1kb)**	Mean base quality**	Coverage**	Busco Complete (n=4104)	BUSCO Single Copy	BUSCO Duplicated	BUSCO Fragmented	BUSCO Missing
A	*Zapus hudsonius*	Meadow jumping mouse	Rodentia	Dipodidae	Least Concern	SAMN07678139	University of Texas Southwestern Medical Center	William Israelsen	Broad Institute	GCA_004024765.1	28,664	32,604	2.24	32.0	61.4	59.7%	58.9%	0.8%	27.5%	12.8%
A	*Glis glis*	Edible dormouse	Rodentia	Gliridae	Least Concern	SAMN07678015	University of California, Los Angeles	Alice Mouton	Uppsala University	GCA_004027185.1	27,095	31,560	2.35	34.9	31.8	49.6%	48.9%	0.7%	33.9%	16.5%
A	*Muscardinus avellanarius*	Hazel dormouse	Rodentia	Gliridae	Least Concern	SAMN07678014	University of California, Los Angeles	Alice Mouton	Uppsala University	GCA_004027005.1	46,810	62,703	2.39	34.8	32.8	68.3%	67.1%	1.2%	23.2%	8.5%
A	*Graphiurus murinus*	Woodland doormouse	Rodentia	Gliridae	Least Concern	SAMN07678085	San Diego Zoo	Oliver Ryder	Uppsala University	GCA_004027655.1	27,731	36,873	2.42	36.2	35.0	51.9%	51.2%	0.7%	29.6%	18.5%
A	*Perognathus longimembris pacificus*	Pacific pocket mouse	Rodentia	Heteromyidae	Least Concern	SAMN07678105	San Diego Zoo	Oliver Ryder	Broad Institute	GCA_004363475.1	27,791	40,656	1.92	34.0	59.6	61.4%	60.5%	0.9%	24.9%	13.7%
A	*Dipodomys stephensi*	Stephen's kangaroo rat	Rodentia	Heteromyidae	Vulnerable	SAMN07678080	San Diego Zoo	Oliver Ryder	Broad Institute	GCA_004024685.1	38,472	45,983	1.95	59.2	59.2	64.7%	63.8%	0.9%	25.5%	9.8%
A	*Hydrochoerus hydrochaeris*	Capybara	Rodentia	Caviidae	Least Concern	SAMN07678143	San Diego Zoo	Oliver Ryder	Uppsala University	GCA_004027455.1	162,656	221,449	2.53	36.9	28.2	86.1%	85.5%	0.6%	9.0%	4.9%
A	*Hystrix cristata*	Northern crested porcupine	Rodentia	Hystricidae	Least Concern	SAMN07678090	San Diego Zoo	Oliver Ryder	Uppsala University	GCA_004026905.1	68,186	77,560	2.13	35.5	35.2	76.7%	76.0%	0.7%	16.1%	7.2%
A	*Acomys cahirinus*	Cairo spiny mouse	Rodentia	Muridae	Least Concern	SAMN07678032	University of Kentucky	Ashley Seifert	Uppsala University	GCA_004027535.1	44,337	68,588	2.20	34.1	32.7	71.2%	70.2%	1.0%	20.0%	8.8%
A	*Meriones unguiculatus*	Mongolian jird	Rodentia	Muridae	Least Concern	SAMN07678137	Harvard Medical School	Vadim Gladyshev	Uppsala University	GCA_004026785.1	81,734	120,489	2.39	36.5	31.3	87.2%	86.6%	0.6%	8.8%	4.0%
A	*Myocastor coypus*	Coypu	Rodentia	Myocastoridae	Least Concern	SAMN07678020	Texas A&M	Bill Murphy	Uppsala University	GCA_004027025.1	35,222	44,764	2.44	34.9	56.5	55.4%	53.8%	1.6%	27.3%	17.3%
A	*Cricetomys gambianus*	Gambian pouched rat	Rodentia	Nesomyidae	Least Concern	SAMN07678025	Cornell University	Danielle Lee	Uppsala University	GCA_004027575.1	100,332	137,544	2.06	33.7	36.7	84.2%	83.7%	0.5%	11.6%	4.2%
A	*Pedetes capensis*	South African springhare	Rodentia	Pedetidae	Least Concern	SAMN07678104	San Diego Zoo	Oliver Ryder	Uppsala University	GCA_007922755.1	15,667	16,472	2.32	34.2	39.7	76.8%	76.0%	0.8%	16.2%	7.0%
A	*Petromus typicus*	Dassie rat	Rodentia	Petromuridae	Least Concern	SAMN07678021	Texas A&M	Bill Murphy	Uppsala University	GCA_004026965.1	34,472	41,518	2.12	34.7	37.7	61.6%	60.9%	0.7%	26.4%	12.0%
A	*Xerus inauris*	Cape ground squirrel	Rodentia	Sciuridae	Least Concern	SAMN07678119	San Diego Zoo	Oliver Ryder	Uppsala University	GCA_004024805.1	70,055	91,124	2.42	35.7	24.4	75.2%	74.2%	1.0%	16.9%	7.9%
A	*Rhizomys pruinosus*	Hoary bamboo rat	Rodentia	Spalacidae	Least Concern	SAMN07678150	The Museum of Vertebrate Zoology at Berkeley	Michael Nachman	Uppsala University	GCA_004026225.1	4,182	4,182	2.58	35.6	25.5	14.5%	13.9%	0.6%	32.1%	53.4%
A	*Thryonomys swinderianus*	Greater cane rat	Rodentia	Thryonomyidae	Least Concern	SAMN07678129	TRACE Wildlife Forensics Network, UK	Rob Ogden	Uppsala University	GCA_004025085.1	25,752	29,412	2.10	35.5	58.2	52.0%	51.4%	0.6%	31.2%	16.8%
A	*Ateles geoffroyi*	Geoffroy's spider monkey	Primates	Atelidae	Endangered	SAMN07678064	San Diego Zoo	Oliver Ryder	Broad Institute	GCA_004024785.1	69,362	82,151	2.45	33.3	60.0	78.2%	77.7%	0.5%	16.1%	5.7%
A	*Alouatta palliata mexicana*	Mexican howler monkey	Primates	Atelidae	Critically Endangered	SAMN07678036	San Diego Zoo	Oliver Ryder	Broad Institute	GCA_004027835.1	59,476	83,839	2.69	33.2	43.1	69.6%	68.0%	1.6%	20.2%	10.2%
A	*Saguinus imperator*	Emperor tamarin	Primates	Cebidae	Least Concern	SAMN07678110	San Diego Zoo	Oliver Ryder	Broad Institute	GCA_004024885.1	68,605	85,066	2.95	33.5	50.1	75.4%	73.7%	1.7%	15.9%	8.7%
A	*Cebus albifrons*	White-fronted capuchin	Primates	Cebidae	Least Concern	SAMN07678068	San Diego Zoo	Oliver Ryder	Broad Institute	GCA_004027755.1	31,522	38,809	2.61	32.7	28.8	53.0%	52.0%	1.0%	30.5%	16.5%
A	*Cercopithecus neglectus*	De brazza's monkey	Primates	Cercopithecidae	Least Concern	SAMN07678070	San Diego Zoo	Oliver Ryder	Broad Institute	GCA_004027615.1	13,610	14,555	2.59	32.2	27.5	40.3%	39.6%	0.7%	32.6%	27.1%
A	*Semnopithecus entellus*	Northern Plains gray langur	Primates	Cercopithecidae	Least Concern	SAMN07678111	San Diego Zoo	Oliver Ryder	Broad Institute	GCA_004025065.1	28,655	34,458	2.70	32.2	29.3	51.6%	50.7%	0.9%	32.2%	16.2%
A	*Erythrocebus patas*	Patas monkey	Primates	Cercopithecidae	Least Concern	SAMN07678082	San Diego Zoo	Oliver Ryder	Broad Institute	GCA_004027335.1	39,816	48,853	2.75	32.7	43.7	62.5%	61.5%	1.0%	25.4%	12.1%
A	*Nasalis larvatus*	Proboscis monkey	Primates	Cercopithecidae	Endangered	SAMN07678099	San Diego Zoo	Oliver Ryder	Broad Institute	GCA_004027105.1	38,381	50,259	2.77	32.5	29.5	59.5%	58.1%	1.4%	26.9%	13.6%
A	*Pygathrix nemaeus*	Red-shanked douc	Primates	Cercopithecidae	Endangered	SAMN07678108	San Diego Zoo	Oliver Ryder	Broad Institute	GCA_004024825.1	60,832	81,292	2.80	33.4	57.9	72.0%	70.8%	1.2%	19.0%	9.0%
A	*Mirza coquereli*	Coquerel's giant mouse lemur	Primates	Cheirogaleidae	Endangered	SAMN07678095	San Diego Zoo	Oliver Ryder	Broad Institute	GCA_004024645.1	64,864	86,037	2.21	32.0	51.7	67.3%	65.9%	1.4%	22.9%	9.8%
A	*Cheirogaleus medius*	Fat-tailed dwarf lemur	Primates	Cheirogaleidae	Least Concern	SAMN07678072	San Diego Zoo	Oliver Ryder	Broad Institute	GCA_004024725.1	98,513	127,877	2.23	32.8	74.6	80.2%	79.0%	1.3%	13.9%	5.9%
A	*Daubentonia madagascariensis*	Aye-aye	Primates	Daubentoniidae	Endangered	SAMN07678077	San Diego Zoo	Oliver Ryder	Broad Institute	GCA_004027145.1	311,553	396,444	2.40	34.3	76.4	91.0%	90.4%	0.6%	6.2%	2.8%
A	*Indri indri*	Indri	Primates	Indridae	Critically Endangered	SAMN07678091	San Diego Zoo	Oliver Ryder	Broad Institute	GCA_004363605.1	33,103	36,269	2.31	32.4	34.4	60.8%	60.0%	0.8%	26.3%	12.9%
A	*Eulemur fulvus*	Common brown lemur	Primates	Lemuridae	Near threatened	SAMN07678083	San Diego Zoo	Oliver Ryder	Broad Institute	GCA_004027275.1	28,544	30,373	2.24	34.0	63.5	52.6%	51.9%	0.7%	31.8%	15.6%
A	*Lemur catta*	Ring tailed lemur	Primates	Lemuridae	Endangered	SAMN07678092	San Diego Zoo	Oliver Ryder	Broad Institute	GCA_004024665.1	77,783	108,458	2.25	31.6	56.0	75.4%	75.0%	0.4%	17.9%	6.7%
A	*Nycticebus coucang*	Sunda slow loris	Primates	Lorisidae	Vulnerable	SAMN07678100	San Diego Zoo	Oliver Ryder	Broad Institute	GCA_004027815.1	23,653	26,596	2.67	32.3	32.3	53.1%	51.6%	1.5%	28.4%	18.5%
A	*Callicebus donacophilus*	White-eared titi	Primates	Pitheciidae	Least Concern	SAMN07678140	San Diego Zoo	Oliver Ryder	Broad Institute	GCA_004027715.1	46,836	52,935	2.64	34.1	23.7	61.2%	60.2%	1.0%	25.5%	13.3%
A	*Pithecia pithecia*	White-faced saki	Primates	Pitheciidae	Least Concern	SAMN07678106	San Diego Zoo	Oliver Ryder	Broad Institute	GCA_004026645.1	70,458	92,467	2.67	33.5	47.3	75.7%	74.8%	0.9%	16.5%	7.8%
A	*Galeopterus variegatus*	Sunda flying lemur	Dermoptera	Cynocephalidae	Least Concern	SAMN07678019	Texas A&M	Bill Murphy	Uppsala University	GCA_004027255.1	44,327	49,765	2.69	36.1	35.5	69.8%	68.9%	0.9%	19.2%	11.0%
A	*Tupaia tana*	Large treeshrew	Scandentia	Tupaiidae	Least Concern	SAMN07678117	San Diego Zoo	Oliver Ryder	Broad Institute	GCA_004365275.1	7,236	7,406	2.28	33.9	25.0	26.6%	26.0%	0.6%	35.6%	37.8%
A	*Chaetophractus vellerosus*	Screaming hairy armadillo	Cingulata	Dasypodidae	Least Concern	SAMN07678071	San Diego Zoo	Oliver Ryder	Uppsala University	GCA_004027955.1	5,322	5,324	2.79	36.2	22.9	15.2%	14.9%	0.3%	34.9%	49.9%
A	*Tolypeutes matacus*	Southern three-banded armadillo	Cingulata	Dasypodidae	Near threatened	SAMN07678115	San Diego Zoo	Oliver Ryder	Uppsala University	GCA_004025125.1	13,475	15,126	3.16	34.0	22.1	38.2%	37.3%	0.9%	33.1%	28.7%
A	*Bradypus variegatus*	Brown-throated sloth	Pilosa	Bradypodidae	Least Concern	SAMN07678054	The Museum of Vertebrate Zoology at Berkeley	Michael Nachman	Broad Institute	GCA_004027775.1	4,994	4,994	0.89	31.7	65.5	20.6%	20.1%	0.5%	20.1%	59.3%
A	*Choloepus didactylus*	Linnaeus's two toed sloth	Pilosa	Megalonychidae	Least Concern	SAMN07678073	San Diego Zoo	Oliver Ryder	Uppsala University	GCA_004027855.1	10,107	10,426	2.59	34.9	19.6	29.3%	28.5%	0.8%	36.1%	34.6%
A	*Myrmecophaga tridactyla*	Giant anteater	Pilosa	Myrmecophagidae	Vulnerable	SAMN07678098	San Diego Zoo	Oliver Ryder	Broad Institute	GCA_004026745.1	44,845	50,817	3.02	32.6	28.9	58.4%	57.8%	0.6%	22.5%	19.1%
A	*Tamandua tetradactyla*	Southern tamandua	Pilosa	Myrmecophagidae	Least Concern	SAMN07678112	San Diego Zoo	Oliver Ryder	Uppsala University	GCA_004025105.1	25,261	27,010	3.13	34.1	30.0	52.1%	50.7%	1.4%	25.2%	22.7%

Supplementary Table 1. Genome assembly statistics for all attemped and successful Zoonomia Project genomes.

Of 173 species and subspecies initially planned for inclusion in the Zoonomia Project, genome assemblies have been generated and released for 132. For the remainder, acquisition of high quality samples was a major impediment. Set A: Discovar only genomes; Set B: Genomes upgraded to longer contiguity; Set C: Assembly failed; Set D: Sample failed QC; Set E: Sample not found; ** Genome assembly statistics output by DISCOVAR *de novo*.

All samples were collected and shipped in compliance with the applicable regulations for sample collection, transfer, export and import.

Set	Species	Common Name	Order	Family	IUCN	Biosample	Provider Institution	Provider contact	Sequencing location	Genbank accession	Contig N50 (bp) **	Scaffold N50 (bp)**	Size (Gb; contigs >1kb)**	Mean base quality**	Coverage**	Busco Complete (n=4104)	BUSCO Single Copy	BUSCO Duplicated	BUSCO Fragmented	BUSCO Missing
A	*Heterohyrax brucei*	African yellow-spotted rock hyrax	Hyracoidea	Procaviidae	Least Concern	SAMN07678088	San Diego Zoo	Oliver Ryder	Uppsala University	GCA_004026845.1	70,066	75,986	3.31	37.2	24.4	76.4%	75.6%	0.8%	15.5%	8.1%
A	*Procavia capensis*	South African rock hyrax	Hyracoidea	Procaviidae	Least Concern	SAMN07678107	San Diego Zoo	Oliver Ryder	Uppsala University	GCA_004026925.1	41,902	44,471	3.34	36.5	23.3	68.3%	67.7%	0.6%	20.8%	10.9%
A	*Chrysochloris asiatica*	Cape golden mole	Afrosoricida	Chrysochloridae	Least Concern	SAMN07678135	University of Stellenbosch , South Africa	TJ Robinson	Broad Institute	GCA_004027935.1	9,805	10,008	3.53	34.0	25.0	32.9%	32.3%	0.6%	33.0%	34.1%
A	*Microgale talazaci*	Talazac's shrew tenrec	Afrosoricida	Tenrecidae	Least Concern	SAMN07678058	The Museum of Vertebrate Zoology at Berkeley	Michael Nachman	Uppsala University	GCA_004026705.1	64,077	73,221	2.96	36.1	27.8	59.8%	59.0%	0.8%	25.8%	14.4%
A	*Elephantulus edwardii*	Cape elephant shrew	Macroscelidea	Macroscelididae	Least Concern	SAMN07678142	University of Stellenbosch , South Africa	TJ Robinson	Broad Institute	GCA_004027355.1	20,663	22,368	3.53	33.1	33.0	48.3%	45.7%	2.6%	30.2%	21.5%
A	*Orycteropus afer*	Aardvark	Tubulidentata	Orycteropodidae	Least Concern	SAMN07678101	San Diego Zoo	Oliver Ryder	Broad Institute	GCA_004365145.1	25,531	28,946	3.90	32.6	35.2	52.1%	51.7%	0.4%	30.4%	17.5%
B	*Diceros bicornis*	Black rhinocerous	Perissodactyla	Rhinocerotidae	Critically Endangered	SAMN07678078				GCA_004027315.2		18,310,000				94.4%	93.9%	0.5%	3.7%	1.9%
B	*Tragulus javanicus*	Java lesser chevrotain	Cetartiodactyla	Tragulidae	Data deficient	SAMN07678116				GCA_004024965.2		18,620,000				91.9%	90.1%	1.8%	4.5%	3.6%
B	*Catagonus wagneri*	Chacoan peccary	Cetartiodactyla	Tayassuidae	Endangered	SAMN07678067				GCA_004024745.2		36,890,000				92.4%	92.0%	0.4%	5.2%	2.4%
B	*Echinops telfairi*	Lesser hedgehog tenrec	Afrosoricida	Tenrecidae	Least Concern	SAMN00216137				GCF_000313985.2		60,390,000				88.2%	87.2%	1.0%	6.5%	5.3%
B	*Antilocapra americana*	Pronghorn	Cetartiodactyla	Antilocapridae	Least Concern	SAMN07678063				GCA_004027515.2		24,010,000				92.8%	90.6%	2.2%	4.1%	3.1%
B	*Galeopterus variegatus*	Sunda flying lemur	Dermoptera	Cynocephalidae	Least Concern	SAMN07678019				GCA_004027255.2		10,140,000				90.4%	89.4%	1.0%	4.8%	4.8%
B	*Procavia capensis*	Rock hyrax	Hyracoidea	Procaviidae	Least Concern	SAMN07678107				GCA_004026925.2		9,110,000				90.5%	89.7%	0.8%	5.1%	4.4%
B	*Hippopotamus amphibius*	Hippopotamus	Cetartiodactyla	Hippopotamidae	Vulnerable	SAMN07678089				GCA_004027065.2		4,860,000				93.2%	91.1%	2.1%	3.9%	2.9%
B	*Moschus moschiferus*	Siberian musk deer	Cetartiodactyla	Moschidae	Vulnerable	SAMN07678096				GCA_004024705.2		32,950,000				92.2%	90.2%	2.0%	4.6%	3.2%
B	*Manis tricuspis*	Tree pangolin	Pholidota	Manidae	Vulnerable	SAMN07678093				GCA_004765945.2		9,930,000				91.0%	90.4%	0.6%	5.0%	4.0%
C	*Neotoma lepida*	Desert woodrat	Rodentia	Cricetidae	Least Concern	SAMN07678052														
C	*Dactylomys boliviensis*	Bolivian bamboo rat	Rodentia	Echimyidae	Least Concern	SAMN07678056														
C	*Rhabdomys pumilio*	Four-striped mouse	Rodentia	Muridae	Least Concern	SAMN07678041														
C	*Tamias amoenus*	Yellow pine chipmunk	Rodentia	Sciuridae	Least Concern	SAMN07678043														
C	*Carlito syrichta*	Philippine tarsier	Primates	Tarsiidae	Near threatened	SAMN07678152														
D	*Procyon lotor*	Raccoon	Carnivora	Procyonidae	Least Concern															
D	*Rhinopoma microphyllum*	Greater mouse-tailed bat	Chiroptera	Rhinopomatidae	Least Concern															
D	*Cyclopes didactylus*	Silky anteater	Pilosa	Cyclopedidae	Least Concern															
D	*Abrocoma bennettii*	Bennett's chinchilla rat	Rodentia	Abrocomidae	Least Concern															
D	*Anomalurus beecrofti*	Beecroft's scaly-tailed squirrel	Rodentia	Anomaluridae	Least Concern															
D	*Laonastes aenigmamus*	Laotian rock rat	Rodentia	Diatomyidae	Least Concern															
D	*Hoplomys gymnurus*	Armored rat	Rodentia	Echimyidae	Least Concern															
D	*Thomomys bottae*	Botta's pocket gopher	Rodentia	Geomyidae	Least Concern															
D	*Chaetodipus spinatus*	Spiny pocket mouse	Rodentia	Heteromyidae	Least Concern															
D	*Mallomys spp.*	Wooly rat	Rodentia	Muridae	Least Concern															
E	*Natalus jamaicensis*	Jamaican greater funnel-eared bat	Chiroptera	Natalidae	Critically Endangered															
E	*Presbytis sp.*	Surili species	Primates	Cercopithecidae	Critically Endangered															
E	*Galea musteloides*	Common yellow-toothed cavy	Rodentia	Caviidae	Data deficient															
E	*Spalax ehrenbergi*	Eastern blind mole rat	Rodentia	Spalacidae	Data deficient															
E	*Amblysomus hottentotus*	Hottentot golden mole	Afrosoricida	Chrysochloridae	Least Concern															
E	*Civettictis civetta*	African civet	Carnivora	Viverridae	Least Concern															
E	*Prionodon pardicolor*	Spotted lingsang	Carnivora	Viverridae	Least Concern															
E	*Saccopteryx bilineata*	Greater sac-winged bat	Chiroptera	Emballonuridae	Least Concern															
E	*Furipterus horrens*	Thumbless bat	Chiroptera	Furipteridae	Least Concern															
E	*Rhinonicteris aurantia*	Orange leaf-nosed bat	Chiroptera	Hipposideridae	Least Concern															
E	*Myzopoda aurita*	Madagascar sucker-footed bat	Chiroptera	Myzopodidae	Least Concern															
E	*Thyroptera tricolor*	Spix's disk-winged bat	Chiroptera	Thyropteridae	Least Concern															
E	*Cistugo seabrae*	Angolan hairy bat	Chiroptera	Vespertilionidae	Least Concern															
E	*Cabassous unicinctus*	Southern naked-tailed armadillo	Cingulata	Dasypodidae	Least Concern															
E	*Cynocephalus volans*	Philippine flying lemur	Dermoptera	Cynocephalidae	Least Concern															

Supplementary Table 1. Genome assembly statistics for all attemped and successful Zoonomia Project genomes.

Of 173 species and subspecies initially planned for inclusion in the Zoonomia Project, genome assemblies have been generated and released for 132. For the remainder, acquisition of high quality samples was a major impediment. Set A: Discovar only genomes; Set B: Genomes upgraded to longer contiguity; Set C: Assembly failed; Set D: Sample failed QC; Set E: Sample not found; ** Genome assembly statistics output by DISCOVAR *de novo*.

All samples were collected and shipped in compliance with the applicable regulations for sample collection, transfer, export and import.

Set	Species	Common Name	Order	Family	IUCN	Biosample	Provider Institution	Provider contact	Sequencing location	Genbank accession	Contig N50 (bp) **	Scaffold N50 (bp)**	Size (Gb; contigs >1kb)**	Mean base quality**	Coverage**	Busco Complete (n=4104)	BUSCO Single Copy	BUSCO Duplicated	BUSCO Fragmented	BUSCO Missing
E	*Echinosorex gymnura*	Moonrat	Eulipotyphla	Erinaceidae	Least Concern															
E	*Rhynchocyon cirnei*	Checkered elephant shrew	Macroscelidea	Macroscelididae	Least Concern															
E	*Calomyscus bailwardi*	Zagros mountains mouse-like hamster	Rodentia	Calomyscidae	Least Concern															
E	*Sicista betulina*	Northern birch mouse	Rodentia	Dipodidae	Least Concern															
E	*Erethizon dorsatum*	North American porcupine	Rodentia	Erethizontidae	Least Concern															
E	*Marmota flaviventris*	Yellow-bellied marmot	Rodentia	Sciuridae	Least Concern															
E	*Tamiasciurus hudsonicus*	American red squirrel	Rodentia	Sciuridae	Least Concern															
E	*Ptilocercus lowii*	Pen-tailed tree shrew	Scandentia	Ptilocercidae	Least Concern															
E	*Bassaricyon neblina*	Olinguito	Carnivora	Procyonidae	Near threatened															
E	*Lepilemur mustelinus*	Weasel sportive lemur	Primates	Lepilemuridae	Near threatened															
E	*Mystacina tuberculata*	New Zealand lesser short-tailed bat	Chiroptera	Mystacinidae	Vulnerable															

Supplementary Table 2.Genomes in Cactus alignment.

A total of 242 genome assemblies, representing 240 species, are included in the Zoonomia Cactus alignment. We included all non-redundant, high-quality assemblies posted on NCBI for >6 months as of March 3, 2018, or for a shorter time if an associated publication was available. One species (dog) is represented by two genomes. Due to a technical error, one genome available on NCBI (Tarsius_syrichta-2.0.1) was not included in this initial alignment, and the genome for Dipodomys stephensi was represented twice.

Order	Family	Species	Common Name	Source	Accession	IUCN*
AFROSORICIDA	Chrysochloridae	*Chrysochloris asiatica*	Cape golden mole	1. Zoonomia	GCA_004027935.1	LC
AFROSORICIDA	Tenrecidae	*Microgale talazaci*	Talazac's shrew tenrec	1. Zoonomia	GCA_004026705.1	LC
CARNIVORA	Canidae	*Canis lupus familiaris*	Domestic dog (village dog)	1. Zoonomia	GCA_004027395.1	LC
CARNIVORA	Canidae	*Vulpes lagopus*	Arctic fox	1. Zoonomia	GCA_004023825.1	LC
CARNIVORA	Eupleridae	*Cryptoprocta ferox*	Fossa	1. Zoonomia	GCA_004023885.1	VU
CARNIVORA	Felidae	*Felis nigripes*	Black-footed cat	1. Zoonomia	GCA_004023925.1	VU
CARNIVORA	Felidae	*Panthera onca*	Jaguar	1. Zoonomia	GCA_004023805.1	NT
CARNIVORA	Herpestidae	*Helogale parvula*	Dwarf mongoose	1. Zoonomia	GCA_004023845.1	LC
CARNIVORA	Herpestidae	*Mungos mungo*	South African banded mongoose	1. Zoonomia	GCA_004023785.1	LC
CARNIVORA	Herpestidae	*Suricata suricatta*	Meerkat	1. Zoonomia	GCA_004023905.1	LC
CARNIVORA	Hyaenidae	*Hyaena hyaena*	Striped hyena	1. Zoonomia	GCA_004023945.1	NT
CARNIVORA	Mephitidae	*Spilogale gracilis*	Western spotted skunk	1. Zoonomia	GCA_004023965.1	LC
CARNIVORA	Mustelidae	*Mellivora capensis*	Honey badger	1. Zoonomia	GCA_004024625.1	LC
CARNIVORA	Mustelidae	*Pteronura brasiliensis*	Giant otter	1. Zoonomia	GCA_004024605.1	EN
CARNIVORA	Otariidae	*Zalophus californianus*	California sea lion	1. Zoonomia	GCA_004024565.1	LC
CARNIVORA	Phocidae	*Mirounga angustirostris*	Northern elephant seal	1. Zoonomia	GCA_004023865.1	LC
CARNIVORA	Viverridae	*Paradoxurus hermaphroditus*	Asian palm civet	1. Zoonomia	GCA_004024585.1	LC
CETARTIODACTYLA	Antilocapridae	*Antilocapra americana*	Pronghorn	1. Zoonomia	GCA_004027515.1	LC
CETARTIODACTYLA	Balaenidae	*Eubalaena japonica*	North Pacific right whale	1. Zoonomia	GCA_004363455.1	EN
CETARTIODACTYLA	Bovidae	*Beatragus hunteri*	Hirola	1. Zoonomia	GCA_004027495.1	CR
CETARTIODACTYLA	Bovidae	*Hemitragus hylocrius*	Nilgiri tahr	1. Zoonomia	GCA_004026825.1	EN
CETARTIODACTYLA	Bovidae	*Ovis canadensis cremnobates*	Peninsular bighorn sheep	1. Zoonomia	GCA_004026945.1	EN
CETARTIODACTYLA	Bovidae	*Saiga tatarica tatarica*	Russian saiga	1. Zoonomia	GCA_004024985.1	CR
CETARTIODACTYLA	Cervidae	*Rangifer tarandus*	Siberian reindeer	1. Zoonomia	GCA_004026565.1	VU
CETARTIODACTYLA	Eschrichtiidae	*Eschrichtius robustus*	Grey whale	1. Zoonomia	GCA_004363415.1	LC
CETARTIODACTYLA	Hippopotamidae	*Hippopotamus amphibius*	Hippopotamus	1. Zoonomia	GCA_004027065.1	VU
CETARTIODACTYLA	Iniidae	*Inia geoffrensis*	Amazon river dolphin	1. Zoonomia	GCA_004363515.1	DD
CETARTIODACTYLA	Monodontidae	*Monodon monoceros*	Narwhal	1. Zoonomia	GCA_004026685.1	LC
CETARTIODACTYLA	Moschidae	*Moschus moschiferus*	Siberian musk deer	1. Zoonomia	GCA_004024705.1	VU
CETARTIODACTYLA	Phocoenidae	*Phocoena phocoena*	Harbor porpoise	1. Zoonomia	GCA_004363495.1	LC
CETARTIODACTYLA	Physeteridae	*Kogia breviceps*	Pygmy sperm whale	1. Zoonomia	GCA_004363705.1	DD
CETARTIODACTYLA	Platanistidae	*Platanista gangetica minor*	Indus river dolphin	1. Zoonomia	GCA_004363435.1	EN
CETARTIODACTYLA	Tayassuidae	*Catagonus wagneri*	Chacoan peccary	1. Zoonomia	GCA_004024745.1	EN
CETARTIODACTYLA	Tragulidae	*Tragulus javanicus*	Java lesser chevrotain	1. Zoonomia	GCA_004024965.1	DD
CETARTIODACTYLA	Ziphiidae	*Mesoplodon bidens*	Sowerby’s beaked whale	1. Zoonomia	GCA_004027085.1	DD
CETARTIODACTYLA	Ziphiidae	*Ziphius cavirostris*	Cuvier's beaked whale	1. Zoonomia	GCA_004364475.1	LC
CHIROPTERA	Craseonycteridae	*Craseonycteris thonglongyai*	Bumblebee bat	1. Zoonomia	GCA_004027555.1	VU
CHIROPTERA	Hipposideridae	*Hipposideros galeritus*	Cantor's leaf-nosed bat	1. Zoonomia	GCA_004027415.1	LC
CHIROPTERA	Megadermatidae	*Megaderma lyra*	Greater false vampire bat	1. Zoonomia	GCA_004026885.1	LC
CHIROPTERA	Molossidae	*Tadarida brasiliensis*	Mexican free-tailed bat	1. Zoonomia	GCA_004025005.1	LC
CHIROPTERA	Mormoopidae	*Mormoops blainvillei*	Ghost-faced bat	1. Zoonomia	GCA_004026545.1	LC
CHIROPTERA	Noctilionidae	*Noctilio leporinus*	Greater bulldog bat	1. Zoonomia	GCA_004026585.1	LC
CHIROPTERA	Phyllostomidae	*Anoura caudifer*	Tailed tailless bat	1. Zoonomia	GCA_004027475.1	LC
CHIROPTERA	Phyllostomidae	*Artibeus jamaicensis*	Jamacian fruit-eating bat	1. Zoonomia	GCA_004027435.1	LC
CHIROPTERA	Phyllostomidae	*Carollia perspicillata*	Seba's short-tailed bat	1. Zoonomia	GCA_004027735.1	LC
CHIROPTERA	Phyllostomidae	*Micronycteris hirsuta*	Hairy big-eared bat	1. Zoonomia	GCA_004026765.1	LC
CHIROPTERA	Phyllostomidae	*Tonatia saurophila*	Stripe-headed round-eared bat	1. Zoonomia	GCA_004024845.1	LC
CHIROPTERA	Pteropodidae	*Macroglossus sobrinus*	Long-tongued fruit bat	1. Zoonomia	GCA_004027375.1	LC
CHIROPTERA	Pteropodidae	*Rousettus aegyptiacus*	Egyptian fruit bat	1. Zoonomia	GCA_004024865.1	LC

Supplementary Table 2.Genomes in Cactus alignment.

A total of 242 genome assemblies, representing 240 species, are included in the Zoonomia Cactus alignment. We included all non-redundant, high-quality assemblies posted on NCBI for >6 months as of March 3, 2018, or for a shorter time if an associated publication was available. One species (dog) is represented by two genomes. Due to a technical error, one genome available on NCBI (Tarsius_syrichta-2.0.1) was not included in this initial alignment, and the genome for Dipodomys stephensi was represented twice.

Order	Family	Species	Common Name	Source	Accession	IUCN*
CHIROPTERA	Vespertilionidae	*Lasiurus borealis*	Eastern red bat	1. Zoonomia	GCA_004026805.1	LC
CHIROPTERA	Vespertilionidae	*Miniopterus schreibersii*	Common bent-wing bat	1. Zoonomia	GCA_004026525.1	NT
CHIROPTERA	Vespertilionidae	*Murina feae*	Ashy-gray tube-nosed bat	1. Zoonomia	GCA_004026665.1	LC
CHIROPTERA	Vespertilionidae	*Myotis myotis*	Greater mouse-eared bat	1. Zoonomia	GCA_004026985.1	LC
CHIROPTERA	Vespertilionidae	*Pipistrellus pipistrellus*	Common pipistrelle	1. Zoonomia	GCA_004026625.1	LC
CINGULATA	Dasypodidae	*Chaetophractus vellerosus*	Screaming hairy armadillo	1. Zoonomia	GCA_004027955.1	LC
CINGULATA	Dasypodidae	*Tolypeutes matacus*	Southern three-banded armadillo	1. Zoonomia	GCA_004025125.1	NT
DERMOPTERA	Cynocephalidae	*Galeopterus variegatus*	Sunda flying lemur	1. Zoonomia	GCA_004027255.1	LC
EULIPOTYPHLA	Solenodontidae	*Solenodon paradoxus*	Hispaniolan solenodon	1. Zoonomia	GCA_004363575.1	EN
EULIPOTYPHLA	Soricidae	*Crocidura indochinensis*	Indochinese shrew	1. Zoonomia	GCA_004027635.1	LC
EULIPOTYPHLA	Talpidae	*Scalopus aquaticus*	Eastern mole	1. Zoonomia	GCA_004024925.1	LC
EULIPOTYPHLA	Talpidae	*Uropsilus gracilis*	Gracile shrew-like mole	1. Zoonomia	GCA_004024945.1	LC
HYRACOIDEA	Procaviidae	*Heterohyrax brucei*	African yellow-spotted rock hyrax	1. Zoonomia	GCA_004026845.1	LC
HYRACOIDEA	Procaviidae	*Procavia capensis*	South African rock hyrax	1. Zoonomia	GCA_004026925.1	LC
LAGOMORPHA	Leporidae	*Lepus americanus*	Snowshoe hare	1. Zoonomia	GCA_004026855.1	LC
MACROSCELIDEA	Macroscelididae	*Elephantulus edwardii*	Cape elephant shrew	1. Zoonomia	GCA_004027355.1	LC
PERISSODACTYLA	Rhinocerotidae	*Ceratotherium simum cottoni*	Northern white rhino	1. Zoonomia	GCA_004027795.1	CR
PERISSODACTYLA	Rhinocerotidae	*Diceros bicornis*	Black rhinocerous	1. Zoonomia	GCA_004027315.1	CR
PERISSODACTYLA	Tapiridae	*Tapirus indicus*	Malayan tapir	1. Zoonomia	GCA_004024905.1	EN
PERISSODACTYLA	Tapiridae	*Tapirus terrestris*	South American tapir	1. Zoonomia	GCA_004025025.1	VU
PILOSA	Megalonychidae	*Choloepus didactylus*	Linnaeus's two toed sloth	1. Zoonomia	GCA_004027855.1	LC
PILOSA	Myrmecophagidae	*Myrmecophaga tridactyla*	Giant anteater	1. Zoonomia	GCA_004026745.1	VU
PILOSA	Myrmecophagidae	*Tamandua tetradactyla*	Southern tamandua	1. Zoonomia	GCA_004025105.1	LC
PRIMATES	Atelidae	*Alouatta palliata mexicana*	Mexican howler monkey	1. Zoonomia	GCA_004027835.1	CR
PRIMATES	Atelidae	*Ateles geoffroyi*	Geoffroy's spider monkey	1. Zoonomia	GCA_004024785.1	EN
PRIMATES	Cebidae	*Cebus albifrons*	White-fronted capuchin	1. Zoonomia	GCA_004027755.1	LC
PRIMATES	Cebidae	*Saguinus imperator*	Emperor tamarin	1. Zoonomia	GCA_004024885.1	LC
PRIMATES	Cercopithecidae	*Cercopithecus neglectus*	De brazza's monkey	1. Zoonomia	GCA_004027615.1	LC
PRIMATES	Cercopithecidae	*Erythrocebus patas*	Patas monkey	1. Zoonomia	GCA_004027335.1	LC
PRIMATES	Cercopithecidae	*Nasalis larvatus*	Proboscis monkey	1. Zoonomia	GCA_004027105.1	EN
PRIMATES	Cercopithecidae	*Pygathrix nemaeus*	Red-shanked douc	1. Zoonomia	GCA_004024825.1	EN
PRIMATES	Cercopithecidae	*Semnopithecus entellus*	Northern Plains gray langur	1. Zoonomia	GCA_004025065.1	LC
PRIMATES	Cheirogaleidae	*Cheirogaleus medius*	Fat-tailed dwarf lemur	1. Zoonomia	GCA_004024725.1	LC
PRIMATES	Cheirogaleidae	*Mirza coquereli*	Coquerel's giant mouse lemur	1. Zoonomia	GCA_004024645.1	EN
PRIMATES	Daubentoniidae	*Daubentonia madagascariensis*	Aye-aye	1. Zoonomia	GCA_004027145.1	EN
PRIMATES	Indridae	*Indri indri*	Indri	1. Zoonomia	GCA_004363605.1	CR
PRIMATES	Lemuridae	*Eulemur fulvus*	Common brown lemur	1. Zoonomia	GCA_004027275.1	NT
PRIMATES	Lemuridae	*Lemur catta*	Ring tailed lemur	1. Zoonomia	GCA_004024665.1	EN
PRIMATES	Lorisidae	*Nycticebus coucang*	Sunda slow loris	1. Zoonomia	GCA_004027815.1	VU
PRIMATES	Pitheciidae	*Callicebus donacophilus*	White-eared titi	1. Zoonomia	GCA_004027715.1	LC
PRIMATES	Pitheciidae	*Pithecia pithecia*	White-faced saki	1. Zoonomia	GCA_004026645.1	LC
RODENTIA	Aplodontiidae	*Aplodontia rufa*	Mountain beaver	1. Zoonomia	GCA_004027875.1	LC
RODENTIA	Capromyidae	*Capromys pilorides*	Desmarest's hutia	1. Zoonomia	GCA_004027915.1	LC
RODENTIA	Castoridae	*Castor canadensis*	North American beaver	1. Zoonomia	GCA_004027675.1	LC
RODENTIA	Caviidae	*Cavia tschudii*	Montane guinea pig	1. Zoonomia	GCA_004027695.1	LC
RODENTIA	Caviidae	*Dolichotis patagonum*	Patagonian mara	1. Zoonomia	GCA_004027295.1	NT
RODENTIA	Caviidae	*Hydrochoerus hydrochaeris*	Capybara	1. Zoonomia	GCA_004027455.1	LC
RODENTIA	Cricetidae	*Ondatra zibethicus*	Muskrat	1. Zoonomia	GCA_004026605.1	LC
RODENTIA	Cricetidae	*Onychomys torridus*	Scorpion mouse	1. Zoonomia	GCA_004026725.1	LC

Supplementary Table 2.Genomes in Cactus alignment.

A total of 242 genome assemblies, representing 240 species, are included in the Zoonomia Cactus alignment. We included all non-redundant, high-quality assemblies posted on NCBI for >6 months as of March 3, 2018, or for a shorter time if an associated publication was available. One species (dog) is represented by two genomes. Due to a technical error, one genome available on NCBI (Tarsius_syrichta-2.0.1) was not included in this initial alignment, and the genome for Dipodomys stephensi was represented twice.

Order	Family	Species	Common Name	Source	Accession	IUCN*
RODENTIA	Cricetidae	*Sigmodon hispidus*	Hispid cotton rat	1. Zoonomia	GCA_004025045.1	LC
RODENTIA	Ctenodactylidae	*Ctenodactylus gundi*	Common gundi	1. Zoonomia	GCA_004027205.1	LC
RODENTIA	Ctenomyidae	*Ctenomys sociabilis*	Social tuco-tuco	1. Zoonomia	GCA_004027165.1	CR
RODENTIA	Cuniculidae	*Cuniculus paca*	Lowland paca	1. Zoonomia	GCA_004365215.1	LC
RODENTIA	Dasyproctidae	*Dasyprocta punctata*	Central American agouti	1. Zoonomia	GCA_004363535.1	LC
RODENTIA	Dinomyidae	*Dinomys branickii*	Pacarana	1. Zoonomia	GCA_004027595.1	LC
RODENTIA	Dipodidae	*Allactaga bullata*	Gobi jerboa	1. Zoonomia	GCA_004027895.1	LC
RODENTIA	Dipodidae	*Zapus hudsonius*	Meadow jumping mouse	1. Zoonomia	GCA_004024765.1	LC
RODENTIA	Gliridae	*Glis glis*	Edible dormouse	1. Zoonomia	GCA_004027185.1	LC
RODENTIA	Gliridae	*Graphiurus murinus*	Woodland doormouse	1. Zoonomia	GCA_004027655.1	LC
RODENTIA	Gliridae	*Muscardinus avellanarius*	Hazel dormouse	1. Zoonomia	GCA_004027005.1	LC
RODENTIA	Heteromyidae	*Dipodomys stephensi*	Stephen's kangaroo rat	1. Zoonomia	GCA_004024685.1	VU
RODENTIA	Heteromyidae	*Perognathus longimembris pacificus*	Pacific pocket mouse	1. Zoonomia	GCA_004363475.1	LC
RODENTIA	Hystricidae	*Hystrix cristata*	Northern crested porcupine	1. Zoonomia	GCA_004026905.1	LC
RODENTIA	Muridae	*Acomys cahirinus*	Cairo spiny mouse	1. Zoonomia	GCA_004027535.1	LC
RODENTIA	Muridae	*Meriones unguiculatus*	Mongolian jird	1. Zoonomia	GCA_004026785.1	LC
RODENTIA	Myocastoridae	*Myocastor coypus*	Coypu	1. Zoonomia	GCA_004027025.1	LC
RODENTIA	Nesomyidae	*Cricetomys gambianus*	Gambian pouched rat	1. Zoonomia	GCA_004027575.1	LC
RODENTIA	Petromuridae	*Petromus typicus*	Dassie rat	1. Zoonomia	GCA_004026965.1	LC
RODENTIA	Sciuridae	*Xerus inauris*	Cape ground squirrel	1. Zoonomia	GCA_004024805.1	LC
RODENTIA	Thryonomyidae	*Thryonomys swinderianus*	Greater cane rat	1. Zoonomia	GCA_004025085.1	LC
SCANDENTIA	Tupaiidae	*Tupaia tana*	Large treeshrew	1. Zoonomia	GCA_004365275.1	LC
TUBULIDENTATA	Orycteropodidae	*Orycteropus afer*	Aardvark	1. Zoonomia	GCA_004365145.1	LC
AFROSORICIDA	Tenrecidae	*Echinops telfairi*	Small madagascar hedgehog	2. Existing assembly	GCF_000313985.1	LC
CARNIVORA	Ailuridae	*Ailurus fulgens*	Lesser panda	2. Existing assembly	GCA_002007465.1	EN
CARNIVORA	Canidae	*Canis lupus familiaris*	Domestic dog	2. Existing assembly	GCF_000002285.3	LC
CARNIVORA	Canidae	*Lycaon pictus*	African hunting dog	2. Existing assembly	GCA_001887905.1	EN
CARNIVORA	Felidae	*Acinonyx jubatus*	Cheetah	2. Existing assembly	GCF_001443585.1	CR
CARNIVORA	Felidae	*Felis catus*	Domestic cat	2. Existing assembly	GCF_000181335.2	LC
CARNIVORA	Felidae	*Panthera pardus*	Leopard	2. Existing assembly	GCA_001857705.1	VU
CARNIVORA	Felidae	*Panthera tigris*	Amur tiger	2. Existing assembly	GCF_000464555.1	EN
CARNIVORA	Felidae	*Puma concolor*	Puma	2. Existing assembly	GCF_003327715.1	LC
CARNIVORA	Mustelidae	*Enhydra lutris*	Sea otter	2. Existing assembly	GCF_002288905.1	EN
CARNIVORA	Mustelidae	*Mustela putorius*	Domestic ferret	2. Existing assembly	GCF_000239315.1	LC
CARNIVORA	Odobenidae	*Odobenus rosmarus*	Pacific walrus	2. Existing assembly	GCF_000321225.1	DD
CARNIVORA	Phocidae	*Leptonychotes weddellii*	Weddell seal	2. Existing assembly	GCF_000349705.1	LC
CARNIVORA	Phocidae	*Neomonachus schauinslandi*	Hawaiian monk seal	2. Existing assembly	GCA_002201575.1	EN
CARNIVORA	Ursidae	*Ailuropoda melanoleuca*	Giant panda	2. Existing assembly	GCA_002007445.1	VU
CARNIVORA	Ursidae	*Ursus maritimus*	Polar bear	2. Existing assembly	GCF_000687225.1	VU
CETARTIODACTYLA	Balaenopteridae	*Balaenoptera acutorostrata*	Minke whale	2. Existing assembly	GCF_000493695.1	LC
CETARTIODACTYLA	Balaenopteridae	*Balaenoptera bonaerensis*	Antarctic minke whale	2. Existing assembly	GCA_000978805.1	DD
CETARTIODACTYLA	Bovidae	*Ammotragus lervia*	Aoudad	2. Existing assembly	GCA_002201775.1	VU
CETARTIODACTYLA	Bovidae	*Bison bison*	American bison	2. Existing assembly	GCF_000754665.1	NT
CETARTIODACTYLA	Bovidae	*Bos indicus*	Zebu cattle	2. Existing assembly	GCA_000247795.2	LC
CETARTIODACTYLA	Bovidae	*Bos mutus*	Wild yak	2. Existing assembly	GCF_000298355.1	VU
CETARTIODACTYLA	Bovidae	*Bos taurus*	Cattle	2. Existing assembly	GCF_000003205.7	LC
CETARTIODACTYLA	Bovidae	*Bubalus bubalis*	Water buffalo	2. Existing assembly	GCF_000471725.1	LC
CETARTIODACTYLA	Bovidae	*Capra aegagrus*	Wild goat	2. Existing assembly	GCA_000978405.1	VU
CETARTIODACTYLA	Bovidae	*Capra hircus*	Goat	2. Existing assembly	GCF_001704415.1	LC

Supplementary Table 2.Genomes in Cactus alignment.

A total of 242 genome assemblies, representing 240 species, are included in the Zoonomia Cactus alignment. We included all non-redundant, high-quality assemblies posted on NCBI for >6 months as of March 3, 2018, or for a shorter time if an associated publication was available. One species (dog) is represented by two genomes. Due to a technical error, one genome available on NCBI (Tarsius_syrichta-2.0.1) was not included in this initial alignment, and the genome for Dipodomys stephensi was represented twice.

Order	Family	Species	Common Name	Source	Accession	IUCN*
CETARTIODACTYLA	Bovidae	*Ovis aries*	Sheep	2. Existing assembly	GCF_000298735.2	LC
CETARTIODACTYLA	Bovidae	*Pantholops hodgsonii*	Chiru	2. Existing assembly	GCF_000400835.1	NT
CETARTIODACTYLA	Camelidae	*Camelus bactrianus*	Bactrian camel	2. Existing assembly	GCF_000767855.1	LC
CETARTIODACTYLA	Camelidae	*Camelus dromedarius*	Arabian camel	2. Existing assembly	GCF_000767585.1	LC
CETARTIODACTYLA	Camelidae	*Camelus ferus*	Wild bactrian camel	2. Existing assembly	GCF_000311805.1	CR
CETARTIODACTYLA	Camelidae	*Vicugna pacos*	Alpaca	2. Existing assembly	GCA_000767525.1	LC
CETARTIODACTYLA	Cervidae	*Elaphurus davidianus*	Pere david's deer	2. Existing assembly	GCA_002443075.1	CR
CETARTIODACTYLA	Cervidae	*Odocoileus virginianus*	White-tailed deer	2. Existing assembly	GCA_002102435.1	LC
CETARTIODACTYLA	Delphinidae	*Orcinus orca*	Killer whale	2. Existing assembly	GCF_000331955.2	DD
CETARTIODACTYLA	Delphinidae	*Tursiops truncatus*	Bottlenose dolphin	2. Existing assembly	GCA_001922835.1	LC
CETARTIODACTYLA	Giraffidae	*Giraffa tippelskirchi*	Giraffe	2. Existing assembly	GCA_001651235.1	VU
CETARTIODACTYLA	Giraffidae	*Okapia johnstoni*	Okapi	2. Existing assembly	GCA_001660835.1	EN
CETARTIODACTYLA	Iniidae	*Lipotes vexillifer*	Yangtze river dolphin	2. Existing assembly	GCF_000442215.1	CR
CETARTIODACTYLA	Monodontidae	*Delphinapterus leucas*	Beluga whale	2. Existing assembly	GCF_002288925.1	LC
CETARTIODACTYLA	Phocoenidae	*Neophocaena asiaeorientalis*	Yangtze finless porpoise	2. Existing assembly	GCA_003031525.1	EN
CETARTIODACTYLA	Suidae	*Sus scrofa*	Pig	2. Existing assembly	GCF_000003025.5	LC
CHIROPTERA	Hipposideridae	*Hipposideros armiger*	Great roundleaf bat	2. Existing assembly	GCA_001890085.1	LC
CHIROPTERA	Mormoopidae	*Pteronotus parnellii*	Parnell's mustached bat	2. Existing assembly	GCA_000465405.1	LC
CHIROPTERA	Phyllostomidae	*Desmodus rotundus*	Common vampire bat	2. Existing assembly	GCA_002940915.2	LC
CHIROPTERA	Pteropodidae	*Eidolon helvum*	Straw-colored fruit bat	2. Existing assembly	GCA_000465285.1	NT
CHIROPTERA	Pteropodidae	*Pteropus alecto*	Black flying fox	2. Existing assembly	GCF_000325575.1	LC
CHIROPTERA	Pteropodidae	*Pteropus vampyrus*	Large flying fox	2. Existing assembly	GCF_000151845.1	NT
CHIROPTERA	Rhinolophidae	*Rhinolophus sinicus*	Chinese rufous horseshoe bat	2. Existing assembly	GCA_001888835.1	LC
CHIROPTERA	Vespertilionidae	*Eptesicus fuscus*	Big brown bat	2. Existing assembly	GCF_000308155.1	LC
CHIROPTERA	Vespertilionidae	*Miniopterus natalensis*	Bats	2. Existing assembly	GCF_001595765.1	LC
CHIROPTERA	Vespertilionidae	*Myotis brandtii*	Brandt's bat	2. Existing assembly	GCF_000412655.1	LC
CHIROPTERA	Vespertilionidae	*Myotis davidii*	Bats	2. Existing assembly	GCF_000327345.1	LC
CHIROPTERA	Vespertilionidae	*Myotis lucifugus*	Little brown bat	2. Existing assembly	GCF_000147115.1	LC
CINGULATA	Dasypodidae	*Dasypus novemcinctus*	Nine-banded armadillo	2. Existing assembly	GCF_000208655.1	LC
EULIPOTYPHLA	Erinaceidae	*Erinaceus europaeus*	Western european hedgehog	2. Existing assembly	GCF_000296755.1	LC
EULIPOTYPHLA	Soricidae	*Sorex araneus*	European shrew	2. Existing assembly	GCF_000181275.1	LC
EULIPOTYPHLA	Talpidae	*Condylura cristata*	Star-nosed mole	2. Existing assembly	GCF_000260355.1	LC
LAGOMORPHA	Leporidae	*Oryctolagus cuniculus*	Rabbit	2. Existing assembly	GCF_000003625.3	NT
LAGOMORPHA	Ochotonidae	*Ochotona princeps*	American pika	2. Existing assembly	GCF_000292845.1	LC
PERISSODACTYLA	Equidae	*Equus asinus*	Ass	2. Existing assembly	GCF_001305755.1	LC
PERISSODACTYLA	Equidae	*Equus caballus*	Horse	2. Existing assembly	GCF_000002305.2	LC
PERISSODACTYLA	Equidae	*Equus przewalskii*	Przewalski's horse	2. Existing assembly	GCF_000696695.1	EN
PERISSODACTYLA	Rhinocerotidae	*Ceratotherium simum*	Southern white rhinoceros	2. Existing assembly	GCF_000283155.1	NT
PERISSODACTYLA	Rhinocerotidae	*Dicerorhinus sumatrensis*	Sumatran rhinoceros	2. Existing assembly	GCA_002844835.1	CR
PHOLIDOTA	Manidae	*Manis javanica*	Malayan pangolin	2. Existing assembly	GCF_001685135.1	CR
PHOLIDOTA	Manidae	*Manis pentadactyla*	Chinese pangolin	2. Existing assembly	GCA_000738955.1	CR
PILOSA	Megalonychidae	*Choloepus hoffmanni*	Hoffmann's two-fingered sloth	2. Existing assembly	GCA_000164785.2	LC
PRIMATES	Aotidae	*Aotus nancymaae*	Ma's night monkey	2. Existing assembly	GCA_000952055.2	VU
PRIMATES	Cebidae	*Callithrix jacchus*	White-tufted-ear marmoset	2. Existing assembly	GCA_002754865.1	LC
PRIMATES	Cebidae	*Cebus capucinus*	White-faced sapajou	2. Existing assembly	GCF_001604975.1	LC
PRIMATES	Cebidae	*Saimiri boliviensis*	Bolivian squirrel monkey	2. Existing assembly	GCF_000235385.1	LC
PRIMATES	Cercopithecidae	*Cercocebus atys*	Sooty mangabey	2. Existing assembly	GCF_000955945.1	NT
PRIMATES	Cercopithecidae	*Chlorocebus sabaeus*	Green monkey	2. Existing assembly	GCF_000409795.2	LC
PRIMATES	Cercopithecidae	*Colobus angolensis*	Angolan colobus	2. Existing assembly	GCF_000951035.1	VU

Supplementary Table 2.Genomes in Cactus alignment.

A total of 242 genome assemblies, representing 240 species, are included in the Zoonomia Cactus alignment. We included all non-redundant, high-quality assemblies posted on NCBI for >6 months as of March 3, 2018, or for a shorter time if an associated publication was available. One species (dog) is represented by two genomes. Due to a technical error, one genome available on NCBI (Tarsius_syrichta-2.0.1) was not included in this initial alignment, and the genome for Dipodomys stephensi was represented twice.

Order	Family	Species	Common Name	Source	Accession	IUCN*
PRIMATES	Cercopithecidae	*Macaca fascicularis*	Crab-eating macaque	2. Existing assembly	GCF_000364345.1	DD
PRIMATES	Cercopithecidae	*Macaca mulatta*	Rhesus monkey	2. Existing assembly	GCF_000772875.2	LC
PRIMATES	Cercopithecidae	*Macaca nemestrina*	Pig-tailed macaque	2. Existing assembly	GCF_000956065.1	VU
PRIMATES	Cercopithecidae	*Mandrillus leucophaeus*	Drill	2. Existing assembly	GCF_000951045.1	EN
PRIMATES	Cercopithecidae	*Papio anubis*	Olive baboon	2. Existing assembly	GCA_000264685.2	LC
PRIMATES	Cercopithecidae	*Piliocolobus tephrosceles*	Ugandan red colobus	2. Existing assembly	GCA_002776525.1	EN
PRIMATES	Cercopithecidae	*Rhinopithecus bieti*	Black snub-nosed monkey	2. Existing assembly	GCF_001698545.1	EN
PRIMATES	Cercopithecidae	*Rhinopithecus roxellana*	Golden snub-nosed monkey	2. Existing assembly	GCF_000769185.1	EN
PRIMATES	Cheirogaleidae	*Microcebus murinus*	Gray mouse lemur	2. Existing assembly	GCA_000165445.3	LC
PRIMATES	Galagidae	*Otolemur garnettii*	Small-eared galago	2. Existing assembly	GCF_000181295.1	LC
PRIMATES	Hominidae	*Gorilla gorilla*	Western lowland gorilla	2. Existing assembly	GCA_900006655.3	CR
PRIMATES	Hominidae	*Homo sapiens*	Human	2. Existing assembly	GCA_000001405.27	LC
PRIMATES	Hominidae	*Pan paniscus*	Pygmy chimpanzee	2. Existing assembly	GCF_000258655.2	EN
PRIMATES	Hominidae	*Pan troglodytes*	Chimpanzee	2. Existing assembly	GCA_002880755.3	EN
PRIMATES	Hominidae	*Pongo abelii*	Sumatran orangutan	2. Existing assembly	GCA_002880775.3	CR
PRIMATES	Hylobatidae	*Nomascus leucogenys*	Northern white-cheeked gibbon	2. Existing assembly	GCF_000146795.2	CR
PRIMATES	Indridae	*Propithecus coquereli*	Coquerel's sifaka	2. Existing assembly	GCF_000956105.1	EN
PRIMATES	Lemuridae	*Eulemur flavifrons*	Sclater's lemur	2. Existing assembly	GCA_001262665.1	CR
PROBOSCIDEA	Elephantidae	*Loxodonta Africana*	African savanna elephant	2. Existing assembly	GCF_000001905.1	VU
RODENTIA	Bathyergidae	*Fukomys damarensis*	Damara mole-rat	2. Existing assembly	GCF_000743615.1	LC
RODENTIA	Bathyergidae	*Heterocephalus glaber*	Naked mole-rat	2. Existing assembly	GCF_000247695.1	LC
RODENTIA	Caviidae	*Cavia aperea*	Brazilian guinea pig	2. Existing assembly	GCA_000688575.1	LC
RODENTIA	Caviidae	*Cavia porcellus*	Domestic guinea pig	2. Existing assembly	GCF_000151735.1	LC
RODENTIA	Chinchillidae	*Chinchilla lanigera*	Long-tailed chinchilla	2. Existing assembly	GCF_000276665.1	EN
RODENTIA	Cricetidae	*Ellobius lutescens*	Transcaucasian mole vole	2. Existing assembly	GCA_001685075.1	LC
RODENTIA	Cricetidae	*Ellobius talpinus*	Northern mole vole	2. Existing assembly	GCA_001685095.1	LC
RODENTIA	Cricetidae	*Mesocricetus auratus*	Golden hamster	2. Existing assembly	GCF_000349665.1	VU
RODENTIA	Cricetidae	*Microtus ochrogaster*	Prairie vole	2. Existing assembly	GCF_000317375.1	LC
RODENTIA	Cricetidae	*Peromyscus maniculatus*	Prairie deer mouse	2. Existing assembly	GCF_000500345.1	LC
RODENTIA	Dipodidae	*Jaculus jaculus*	Lesser egyptian jerboa	2. Existing assembly	GCF_000280705.1	LC
RODENTIA	Heteromyidae	*Dipodomys ordii*	Ord's kangaroo rat	2. Existing assembly	GCF_000151885.1	LC
RODENTIA	Muridae	*Mus caroli*	Ryukyu mouse	2. Existing assembly	GCA_900094665.2	LC
RODENTIA	Muridae	*Mus musculus*	House mouse	2. Existing assembly	GCF_000001635.26	LC
RODENTIA	Muridae	*Mus pahari*	Shrew mouse	2. Existing assembly	GCA_900095145.2	LC
RODENTIA	Muridae	*Mus spretus*	Western wild mouse	2. Existing assembly	GCA_001624865.1	LC
RODENTIA	Muridae	*Psammomys obesus*	Fat sand rat	2. Existing assembly	GCA_002215935.1	LC
RODENTIA	Muridae	*Rattus norvegicus*	Norway rat	2. Existing assembly	GCF_000001895.5	LC
RODENTIA	Nesomyidae	*Cricetulus griseus*	Chinese hamster	2. Existing assembly	GCA_900186095.1	LC
RODENTIA	Octodontidae	*Octodon degus*	Degu	2. Existing assembly	GCF_000260255.1	LC
RODENTIA	Sciuridae	*Ictidomys tridecemlineatus*	Thirteen-lined ground squirrel	2. Existing assembly	GCF_000236235.1	LC
RODENTIA	Sciuridae	*Marmota marmota*	Alpine marmot	2. Existing assembly	GCF_001458135.1	LC
RODENTIA	Sciuridae	*Spermophilus dauricus*	Daurian ground squirrel	2. Existing assembly	GCA_002406435.1	LC
RODENTIA	Spalacidae	*Nannospalax galili*	Upper galilee mountains blind mole rat	2. Existing assembly	GCF_000622305.1	DD
SCANDENTIA	Tupaiidae	*Tupaia belangeri chinensis*	Northern tree shrew	2. Existing assembly	GCF_000334495.1	LC
SIRENIA	Trichechidae	*Trichechus manatus*	Florida manatee	2. Existing assembly	GCF_000243295.1	EN
CETARTIODACTYLA	Iniidae	*Pontoporia blainvillei*	La plata dolphin	3. Zoonomia (not in alignment)	GCA_004363935.1	VU
CHIROPTERA	Phyllostomidae	*Macrotus californicus*	California leaf-nosed bat	3. Zoonomia (not in alignment)	GCA_007922815.1	LC
CHIROPTERA	Rhinolophidae	*Rhinolophus ferrumequinum*	Greater horseshoe bat	3. Zoonomia (not in alignment)	GCA_007922735.1	LC
CHIROPTERA	Vespertilionidae	*Antrozous pallidus*	Pallid bat	3. Zoonomia (not in alignment)	GCA_007922775.1	LC

Supplementary Table 2.Genomes in Cactus alignment.

A total of 242 genome assemblies, representing 240 species, are included in the Zoonomia Cactus alignment. We included all non-redundant, high-quality assemblies posted on NCBI for >6 months as of March 3, 2018, or for a shorter time if an associated publication was available. One species (dog) is represented by two genomes. Due to a technical error, one genome available on NCBI (Tarsius_syrichta-2.0.1) was not included in this initial alignment, and the genome for Dipodomys stephensi was represented twice.

Order	Family	Species	Common Name	Source	Accession	IUCN*
CHIROPTERA	Vespertilionidae	*Nycticeius humeralis*	Egyptian slit-faced bat	3. Zoonomia (not in alignment)	GCA_007922795.1	LC
PHOLIDOTA	Manidae	*Manis tricuspis*	Tree pangolin	3. Zoonomia (not in alignment)	GCA_004765945.1	VU
PILOSA	Bradypodidae	*Bradypus variegatus*	Brown-throated sloth	3. Zoonomia (not in alignment)	GCA_004027775.1	LC
RODENTIA	Pedetidae	*Pedetes capensis*	South African springhare	3. Zoonomia (not in alignment)	GCA_007922755.1	LC
RODENTIA	Spalacidae	*Rhizomys pruinosus*	Hoary bamboo rat	3. Zoonomia (not in alignment)	GCA_004026225.1	LC

* LC = Least Concern; NT = Near threatened; VU = Vulnerable; EN = Endangered; CR = Critically endangered

Supplementary Table 3. Genetic diversity in Zoonomia genome assemblies (source data for figure 2)

N	Species	Common Name	IUCN	population	In diversity statistical analysis?	% genome callable	allelic balance (ab)	Overall hetero-zygosity	Segments of homo-zygosity (SoH)	% genome callable; upgraded	allelic balance (ab); upgraded	Overall hetero-zygosity; upgraded	Segments of homo-zygosity (SoH); upgraded
1	*Solenodon paradoxus*	Hispaniolan solenodon	Endangered	wild	yes	97.6%	0.211	0.0010	35.9%				
2	*Crocidura indochinensis*	Indochinese shrew	Least Concern	unknown	yes	93.2%	0.274	0.0049	8.5%				
3	*Scalopus aquaticus*	Eastern mole	Least Concern	wild	yes	98.1%	0.236	0.0008	81.3%				
4	*Uropsilus gracilis*	Gracile shrew-like mole	Least Concern	wild	yes	97.4%	0.209	0.0020	20.4%				
5	*Vulpes lagopus*	Arctic fox	Least Concern	captive	yes	96.8%	0.188	0.0016	49.6%				
6	*Canis lupus familiaris*	Domestic dog (village dog)	Least Concern	wild	yes	96.8%	0.181	0.0021	26.2%				
7	*Cryptoprocta ferox*	Fossa	Vulnerable	captive	yes	98.3%	0.228	0.0015	11.1%				
8	*Felis nigripes*	Black-footed cat	Vulnerable	captive	no (high ab)	86.4%	0.383	0.0008	41.5%				
9	*Panthera onca*	Jaguar	Near Threatened	captive	yes	96.7%	0.232	0.0009	51.7%				
10	*Helogale parvula*	Dwarf mongoose	Least Concern	captive	yes	97.4%	0.199	0.0012	53.0%				
11	*Suricata suricatta*	Meerkat	Least Concern	captive	yes	97.7%	0.135	0.0036	9.4%				
12	*Mungos mungo*	South African banded mongoose	Least Concern	wild	yes	97.6%	0.145	0.0025	10.3%				
13	*Hyaena hyaena*	Striped hyena	Near Threatened	captive	yes	97.4%	0.288	0.0011	27.7%				
14	*Spilogale gracilis*	Western spotted skunk	Least Concern	wild	yes	96.8%	0.192	0.0014	43.0%				
15	*Pteronura brasiliensis*	Giant otter	Endangered	wild	yes	98.3%	0.262	0.0011	39.8%				
16	*Mellivora capensis*	Honey badger	Least Concern	wild	yes	97.6%	0.172	0.0034	11.1%				
17	*Zalophus californianus*	California sea lion	Least Concern	wild	yes	94.6%	0.252	0.0009	35.4%				
18	*Mirounga angustirostris*	Northern elephant seal	Least Concern	wild	no (high ab)	98.3%	0.396	0.0004	58.8%				
19	*Paradoxurus hermaphroditus*	Asian palm civet	Least Concern	wild	yes	96.9%	0.153	0.0042	16.5%				
20	*Manis tricuspis*	Tree pangolin	Vulnerable	wild	failed (unknown) *								
21	*Diceros bicornis*	Black rhinocerous	Critically Endangered	wild	yes	98.4%	0.208	0.0019	18.2%	98.2%	0.209	0.0019	17.4%
22	*Ceratotherium simum cottoni*	Northern white rhino	Critically Endangered	wild	no (high ab)	90.7%	0.361	0.0012	42.9%				
23	*Tapirus indicus*	Malayan tapir	Endangered	captive	yes	99.1%	0.211	0.0009	50.8%				
24	*Tapirus terrestris*	South American tapir	Vulnerable	captive	yes	98.2%	0.137	0.0035	7.5%				
25	*Antilocapra americana*	Pronghorn	Least Concern	captive	yes	98.5%	0.225	0.0015	48.0%	98.6%	0.225	0.0015	48.0%
26	*Eubalaena japonica*	North Pacific right whale	Endangered	wild	yes	97.6%	0.264	0.0019	17.7%				
27	*Beatragus hunteri*	Hirola	Critically Endangered	captive	no (high ab)	95.6%	0.388	0.0005	59.5%				
28	*Hemitragus hylocrius*	Nilgiri tahr	Endangered	unknown	no (high ab)	97.1%	0.411	0.0004	76.0%				
29	*Ovis canadensis cremnobates*	Peninsular bighorn sheep	Endangered	unknown	yes	95.3%	0.328	0.0008	39.2%				
30	*Saiga tatarica tatarica*	Russian saiga	Critically Endangered	captive	no (high ab)	87.5%	0.456	0.0025	30.1%				
31	*Rangifer tarandus*	Siberian reindeer	Vulnerable	unknown	yes	98.0%	0.163	0.0030	8.7%				
32	*Eschrichtius robustus*	Grey whale	Least Concern	wild	yes	98.1%	0.288	0.0008	28.4%				
33	*Hippopotamus amphibius*	Hippopotamus	Vulnerable	captive	yes	93.4%	0.064	0.0033	19.3%	93.0%	0.063	0.0031	18.1%
34	*Inia geoffrensis*	Amazon river dolphin	Data deficient	wild	yes	92.8%	0.350	0.0009	32.5%				
35	*Kogia breviceps*	Pygmy sperm whale	Data deficient	wild	yes	95.8%	0.145	0.0059	1.5%				
36	*Monodon monoceros*	Narwhal	Least Concern	wild	yes	97.7%	0.242	0.0010	NA				
38	*Moschus moschiferus*	Siberian musk deer	Vulnerable	captive	yes	97.8%	0.214	0.0017	31.5%	97.6%	0.213	0.0016	31.5%
39	*Phocoena phocoena*	Harbor porpoise	Least Concern	wild	yes	98.2%	0.204	0.0020	8.4%				
40	*Platanista gangetica minor*	Indus river dolphin	Endangered	wild	no (high ab)	95.1%	0.551	0.0005	69.7%				
41	*Pontoporia blainvillei*	La plata dolphin	Vulnerable	wild	no (high ab)	83.8%	0.498	0.0021	NA				
42	*Catagonus wagneri*	Chacoan peccary	Endangered	captive	no (high ab)	85.7%	0.561	0.0041	NA	85.7%	0.561	0.0041	NA
43	*Tragulus javanicus*	Java lesser chevrotain	Data deficient	captive	yes	96.0%	0.110	0.0047	16.1%	95.5%	0.111	0.0047	18.1%
44	*Ziphius cavirostris*	Cuvier's beaked whale	Least Concern	wild	yes	89.1%	0.329	0.0051	NA				
45	*Mesoplodon bidens*	Sowerby’s beaked whale	Data deficient	wild	yes	95.2%	0.269	0.0021	1.7%				
46	*Craseonycteris thonglongyai*	Bumblebee bat	Vulnerable	wild	yes	89.1%	0.207	0.0039	16.1%				
47	*Hipposideros galeritus*	Cantor's leaf-nosed bat	Least Concern	wild	yes	94.8%	0.188	0.0046	10.5%				
48	*Megaderma lyra*	Greater false vampire bat	Least Concern	wild	yes	97.8%	0.235	0.0024	14.6%				
49	*Tadarida brasiliensis*	Mexican free-tailed bat	Least Concern	wild	yes	92.1%	0.117	0.0067	NA				

Supplementary Table 3. Genetic diversity in Zoonomia genome assemblies (source data for figure 2)

N	Species	Common Name	IUCN	population	In diversity statistical analysis?	% genome callable	allelic balance (ab)	Overall hetero-zygosity	Segments of homo-zygosity (SoH)	% genome callable; upgraded	allelic balance (ab); upgraded	Overall hetero-zygosity; upgraded	Segments of homo-zygosity (SoH); upgraded
50	*Mormoops blainvillei*	Ghost-faced bat	Least Concern	wild	yes	97.6%	0.137	0.0041	5.8%				
51	*Noctilio leporinus*	Greater bulldog bat	Least Concern	wild	yes	98.8%	0.240	0.0004	NA				
52	*Macrotus californicus*	California leaf-nosed bat	Least Concern	wild	yes	93.3%	0.145	0.0023	5.9%				
53	*Micronycteris hirsuta*	Hairy big-eared bat	Least Concern	wild	yes	97.2%	0.122	0.0046	15.8%				
54	*Artibeus jamaicensis*	Jamacian fruit-eating bat	Least Concern	wild	yes	94.3%	0.141	0.0046	26.5%				
55	*Carollia perspicillata*	Seba's short-tailed bat	Least Concern	wild	yes	88.7%	0.210	0.0076	2.5%				
56	*Tonatia saurophila*	Stripe-headed round-eared bat	Least Concern	wild	yes	98.3%	0.154	0.0030	11.0%				
57	*Anoura caudifer*	Tailed tailless bat	Least Concern	wild	yes	98.0%	0.158	0.0022	11.3%				
58	*Rousettus aegyptiacus*	Egyptian fruit bat	Least Concern	wild	yes	96.7%	0.141	0.0025	45.0%				
59	*Macroglossus sobrinus*	Long-tongued fruit bat	Least Concern	wild	yes	97.7%	0.143	0.0026	26.3%				
60	*Rhinolophus ferrumequinum*	Greater horseshoe bat	Least Concern	wild	no (outlier)	52.8%	0.156	0.0251	NA				
61	*Murina feae*	Ashy-gray tube-nosed bat	Least Concern	wild	yes	95.4%	0.172	0.0033	7.6%				
62	*Miniopterus schreibersii*	Common bent-wing bat	Near Threatened	wild	yes	95.3%	0.278	0.0009	37.0%				
63	*Pipistrellus pipistrellus*	Common pipistrelle	Least Concern	wild	yes	95.5%	0.126	0.0036	11.7%				
64	*Lasiurus borealis*	Eastern red bat	Least Concern	wild	yes	95.8%	0.100	0.0063	40.0%				
65	*Nycticeius humeralis*	Egyptian slit-faced bat	Least Concern	wild	no (outlier)	51.3%	0.151	0.0240	2.8%				
66	*Myotis myotis*	Greater mouse-eared bat	Least Concern	wild	yes	96.9%	0.226	0.0033	7.4%				
67	*Antrozous pallidus*	Pallid bat	Least Concern	wild	yes	95.8%	0.165	0.0017	56.1%				
68	*Lepus Americanus*	Snowshoe hare	Least Concern	unknown	yes	94.1%	0.217	0.0040	7.9%				
69	*Aplodontia rufa*	Mountain beaver	Least Concern	wild	yes	94.6%	0.294	0.0029	8.7%				
70	*Capromys pilorides*	Desmarest's hutia	Least Concern	wild	yes	91.0%	0.292	0.0050	41.2%				
71	*Castor canadensis*	North American beaver	Least Concern	wild	yes	97.0%	0.262	0.0016	20.6%				
72	*Cavia tschudii*	Montane guinea pig	Least Concern	captive	yes	96.6%	0.338	0.0015	64.1%				
73	*Dolichotis patagonum*	Patagonian mara	Near Threatened	captive	yes	94.2%	0.191	0.0041	41.1%				
74	*Sigmodon hispidus*	Hispid cotton rat	Least Concern	captive	no (high ab)	98.5%	0.495	0.0004	NA				
75	*Ondatra zibethicus*	Muskrat	Least Concern	wild	yes	98.0%	0.168	0.0019	47.3%				
76	*Onychomys torridus*	Scorpion mouse	Least Concern	wild	yes	96.4%	0.268	0.0035	15.8%				
77	*Ctenodactylus gundi*	Common gundi	Least Concern	wild	yes	98.1%	0.178	0.0023	8.7%				
78	*Ctenomys sociabilis*	Social tuco-tuco	Critically Endangered	lab	yes	95.3%	0.335	0.0006	78.7%				
79	*Cuniculus paca*	Lowland paca	Least Concern	wild	yes	88.9%	0.297	0.0070	20.3%				
80	*Dasyprocta punctata*	Central American agouti	Least Concern	captive	yes	94.0%	0.108	0.0050	11.0%				
81	*Dinomys branickii*	Pacarana	Least Concern	unknown	yes	97.3%	0.332	0.0006	59.0%				
82	*Allactaga bullata*	Gobi jerboa	Least Concern	wild	yes	94.6%	0.228	0.0033	NA				
83	*Zapus hudsonius*	Meadow jumping mouse	Least Concern	lab	yes	91.3%	0.196	0.0045	6.7%				
84	*Glis glis*	Edible dormouse	Least Concern	wild	no (high ab)	95.8%	0.770	0.0008	NA				
85	*Muscardinus avellanarius*	Hazel dormouse	Least Concern	wild	no (high ab)	96.9%	0.399	0.0004	NA				
86	*Graphiurus murinus*	Woodland doormouse	Least Concern	captive	yes	94.9%	0.165	0.0026	69.7%				
87	*Perognathus longimembris pacificus*	Pacific pocket mouse	Least Concern	wild	yes	97.0%	0.17	0.0026	0.35				
88	*Dipodomys stephensi*	Stephen's kangaroo rat	Vulnerable	unknown	no (high ab)	91.5%	0.468	0.0020	28.2%				
89	*Hydrochoerus hydrochaeris*	Capybara	Least Concern	wild	yes	97.5%	0.273	0.0012	19.1%				
90	*Hystrix cristata*	Northern crested porcupine	Least Concern	captive	yes	96.5%	0.152	0.0043	1.9%				
91	*Acomys cahirinus*	Cairo spiny mouse	Least Concern	lab	no (high ab)	97.4%	0.425	0.0002	NA				
92	*Meriones unguiculatus*	Mongolian jird	Least Concern	captive	yes	96.8%	0.269	0.0008	74.3%				
93	*Myocastor coypus*	Coypu	Least Concern	wild	yes	97.6%	0.256	0.0017	24.7%				
94	*Cricetomys gambianus*	Gambian pouched rat	Least Concern	wild	yes	97.2%	0.236	0.0022	18.7%				
95	*Pedetes capensis*	South African springhare	Least Concern	captive	yes	94.3%	0.110	0.0064	3.2%				
96	*Petromus typicus*	Dassie rat	Least Concern	wild	yes	95.4%	0.120	0.0043	13.9%				
97	*Xerus inauris*	Cape ground squirrel	Least Concern	wild	yes	94.9%	0.231	0.0017	35.0%				

Supplementary Table 3. Genetic diversity in Zoonomia genome assemblies (source data for figure 2)

N	Species	Common Name	IUCN	population	In diversity statistical analysis?	% genome callable	allelic balance (ab)	Overall hetero-zygosity	Segments of homo-zygosity (SoH)	% genome callable; upgraded	allelic balance (ab); upgraded	Overall hetero-zygosity; upgraded	Segments of homo-zygosity (SoH); upgraded
98	*Rhizomys pruinosus*	Hoary bamboo rat	Least Concern	wild	failed (short contiguity)								
99	*Thryonomys swinderianus*	Greater cane rat	Least Concern	wild	yes	96.9%	0.178	0.0036	8.5%				
100	*Ateles geoffroyi*	Geoffroy's spider monkey	Endangered	wild	no (high ab)	88.4%	0.417	0.0038	6.4%				
101	*Alouatta palliata mexicana*	Mexican howler monkey	Critically Endangered	captive	yes	98.4%	0.298	0.0010	68.3%				
102	*Saguinus imperator*	Emperor tamarin	Least Concern	captive	yes	98.0%	0.205	0.0031	15.3%				
103	*Cebus albifrons*	White-fronted capuchin	Least Concern	captive	yes	93.8%	0.272	0.0020	17.7%				
104	*Cercopithecus neglectus*	De brazza's monkey	Least Concern	captive	yes	91.9%	0.342	0.0023	28.1%				
105	*Semnopithecus entellus*	Northern Plains gray langur	Least Concern	captive	yes	95.0%	0.239	0.0023	20.9%				
106	*Erythrocebus patas*	Patas monkey	Least Concern	captive	yes	96.9%	0.245	0.0023	NA				
107	*Nasalis larvatus*	Proboscis monkey	Endangered	captive	yes	96.8%	0.256	0.0014	40.9%				
108	*Pygathrix nemaeus*	Red-shanked douc	Endangered	captive	yes	97.9%	0.192	0.0019	24.0%				
109	*Mirza coquereli*	Coquerel's giant mouse lemur	Endangered	captive	no (high ab)	88.2%	0.491	0.0037	10.6%				
110	*Cheirogaleus medius*	Fat-tailed dwarf lemur	Least Concern	captive	no (high ab)	87.4%	0.427	0.0024	26.9%				
111	*Daubentonia madagascariensis*	Aye-aye	Endangered	wild	yes	97.7%	0.250	0.0007	43.2%				
112	*Indri indri*	Indri	Critically Endangered	wild	yes	95.0%	0.134	0.0051	23.2%				
113	*Eulemur fulvus*	Common brown lemur	Near Threatened	captive	yes	96.6%	0.125	0.0062	NA				
114	*Lemur catta*	Ring tailed lemur	Endangered	captive	yes	94.1%	0.130	0.0034	19.9%				
115	*Nycticebus coucang*	Sunda slow loris	Vulnerable	wild	yes	94.6%	0.294	0.0026	25.2%				
116	*Callicebus donacophilus*	White-eared titi	Least Concern	wild	yes	94.8%	0.313	0.0017	29.5%				
117	*Pithecia pithecia*	White-faced saki	Least Concern	wild	yes	98.3%	0.191	0.0018	33.3%				
118	*Galeopterus variegatus*	Sunda flying lemur	Least Concern	wild	yes	95.4%	0.164	0.0045	30.3%	95.1%	0.164	0.0043	30.3%
119	*Tupaia tana*	Large treeshrew	Least Concern	captive	yes	88.8%	0.305	0.0060	23.8%				
120	*Chaetophractus vellerosus*	Screaming hairy armadillo	Least Concern	wild	failed (short contiguity)								
121	*Tolypeutes matacus*	Southern three-banded armadillo	Near Threatened	captive	yes	86.1%	0.333	0.0042	11.0%				
122	*Bradypus variegatus*	Brown-throated sloth	Least Concern	wild	no (high ab)	96.3%	0.49	0.0116	0.04				
123	*Choloepus didactylus*	Linnaeus's two toed sloth	Least Concern	wild	no (high ab)	87.2%	0.388	0.0037	35.6%				
124	*Myrmecophaga tridactyla*	Giant anteater	Vulnerable	captive	yes	95.4%	0.243	0.0035	3.9%				
125	*Tamandua tetradactyla*	Southern tamandua	Least Concern	captive	yes	88.6%	0.166	0.0056	8.1%				
126	*Heterohyrax brucei*	African yellow-spotted rock hyrax	Least Concern	captive	yes	94.9%	0.159	0.0047	4.9%				
127	*Procavia capensis*	South African rock hyrax	Least Concern	wild	yes	93.7%	0.145	0.0058	6.4%	94.7%	0.145	0.0054	8.2%
128	*Chrysochloris asiatica*	Cape golden mole	Least Concern	wild	failed (short contiguity)								
130	*Microgale talazaci*	Talazac's shrew tenrec	Least Concern	wild	yes	95.8%	0.161	0.0047	9.0%				
131	*Elephantulus edwardii*	Cape elephant shrew	Least Concern	wild	yes	95.8%	0.257	0.0032	12.7%				
132	*Orycteropus afer*	Aardvark	Least Concern	wild	yes	96.6%	0.295	0.0021	17.1%				

* failed for undetermined reasons
